# Effect of Packaging Materials on Lettuce (*Lactuca sativa* var. *capitata*) Chemical Quality Under Cold Storage

**DOI:** 10.1155/ijfo/9968381

**Published:** 2026-07-02

**Authors:** Vu Thi Kim Oanh, Tolcha Techane Alemu

**Affiliations:** ^1^ Department of Post-Harvest Technology, Vietnam National University of Agriculture, Hanoi, Vietnam, vnua.edu.vn; ^2^ Department of Post-Harvest Management, Jimma University College of Agriculture and Veterinary Medicine, Jimma, Ethiopia

**Keywords:** iceberg lettuce, packaging materials, quality

## Abstract

Lettuce is a perishable vegetable with a short shelf life, which leads to detrimental changes in texture, physiological processes, and enzymatic activity during postharvest storage. However, appropriate packaging materials can be used in storage and distribution to maintain quality and extend the shelf life. Therefore, the aim of this study was to investigate the effect of different packaging materials on lettuce chemical quality under cold storage. The experimental analysis was conducted at intervals of every 6 days for 33 days with three replications and evaluated in a CRD trial corresponding to nine packaging materials. The samples were harvested at 58 DAP (days after planting). In this study, nine packaging types were evaluated: (1) without primary packaging in a carton box, (2) a carton box glued with low‐density polyethylene (LDPE), and (3) a Danpla plastic box; (4) samples wrapped in LDPE and (5) green modified atmosphere packaging separately; and (6) samples packed in a carton box, (7) a carton box glued with LDPE film, and (8) a Danpla plastic box. Quality parameters (TSS, vitamin C, phenolics, total chlorophylls, and carotenoids) were analyzed. This work concludes that the iceberg lettuce “Saula” packed in a carton box glued with LDPE stored at 3^°^C ± 2^°^C and 95% relative humidity prolongs the shelf life and is, therefore, recommended for exportation.

## 1. Introduction

Iceberg lettuce (*Lactuca sativa* var. *capitata*) is one of the most important leafy vegetables, mostly consumed fresh, especially in salads [1, 2]. However, it is highly perishable due to rapid physiological, biochemical, and enzymatic changes that occur after harvest, leading to quality degradation and a shortened shelf life [3]. Lettuce contains a lot of bioactive components like vitamins, minerals, phytochemicals, and dietary fiber, which contribute to health benefits [4, 5]. This vegetable also has a great role in economic growth, particularly in marketing systems like exportation [6]. Therefore, maintaining the postharvest quality is essential for export markets characterized by longer storage and transport durations. The application of appropriate packaging materials could potentially extend the shelf life of lettuce by managing various factors such as environmental, physical, and physiological factors [4, 7, 8].

Eriksson et al. [9] reported that vegetables are the most lost produce at retail stores, and iceberg lettuce is the third most lost. Lam [10] reported that in Vietnam, 25%–30% quantitative and qualitative PHL of vegetables is due to weakness in the postharvest management system. Iceberg lettuce loss of 20%–30% is due to mechanical stress [11]. The export of iceberg lettuce to international and neighboring markets by air freight has decreased due to increased competition and the high cost of air freight. Transportation by sea freight is half of that by air freight; this may take time, and as a result, the quality of lettuce is affected [12–14]. Ikegaya et al. [13] also reported that the physicochemical properties of lettuce are affected during export by sea. Packaging plays a crucial role in preserving the quality of fresh vegetables by minimizing moisture loss, reducing mechanical damage, and delaying senescence [14, 15]. Selecting appropriate packaging materials can significantly influence the retention of key chemical quality attributes such as total soluble solids (TSSs), vitamin C, total phenolics, chlorophylls, and carotenoids during storage.

The studies of postharvest properties of lettuce for export purposes have been limited to chemical quality parameters in relation to the different packaging materials. While various packaging materials have been explored, limited information is available on their comparative effectiveness under cold storage conditions. These packaging materials play a great role in maintaining quality and extending shelf life by controlling temperature and relative humidity. To the best of our knowledge, there is not enough information on the effect of packaging materials on the chemical quality parameters of iceberg lettuce under cold storage for export. The findings of this study will help identify the most suitable packaging approach to extend shelf life and support export potential. Therefore, this study was planned to evaluate the effect of packaging on the chemical quality parameters of iceberg lettuce during cold storage to suggest the most suitable packaging materials for export.

## 2. Materials and Methods

The experiments were conducted in the laboratory of the Department of Postharvest Technology, Faculty of Food Science and Technology, Vietnam National University of Agriculture (VNUA) in Gia Lam, Hanoi (N 21°, E 105.93°). This study was conducted from February to December 2023.

### 2.1. Materials

#### 2.1.1. Plant Materials

**Figure 1:**
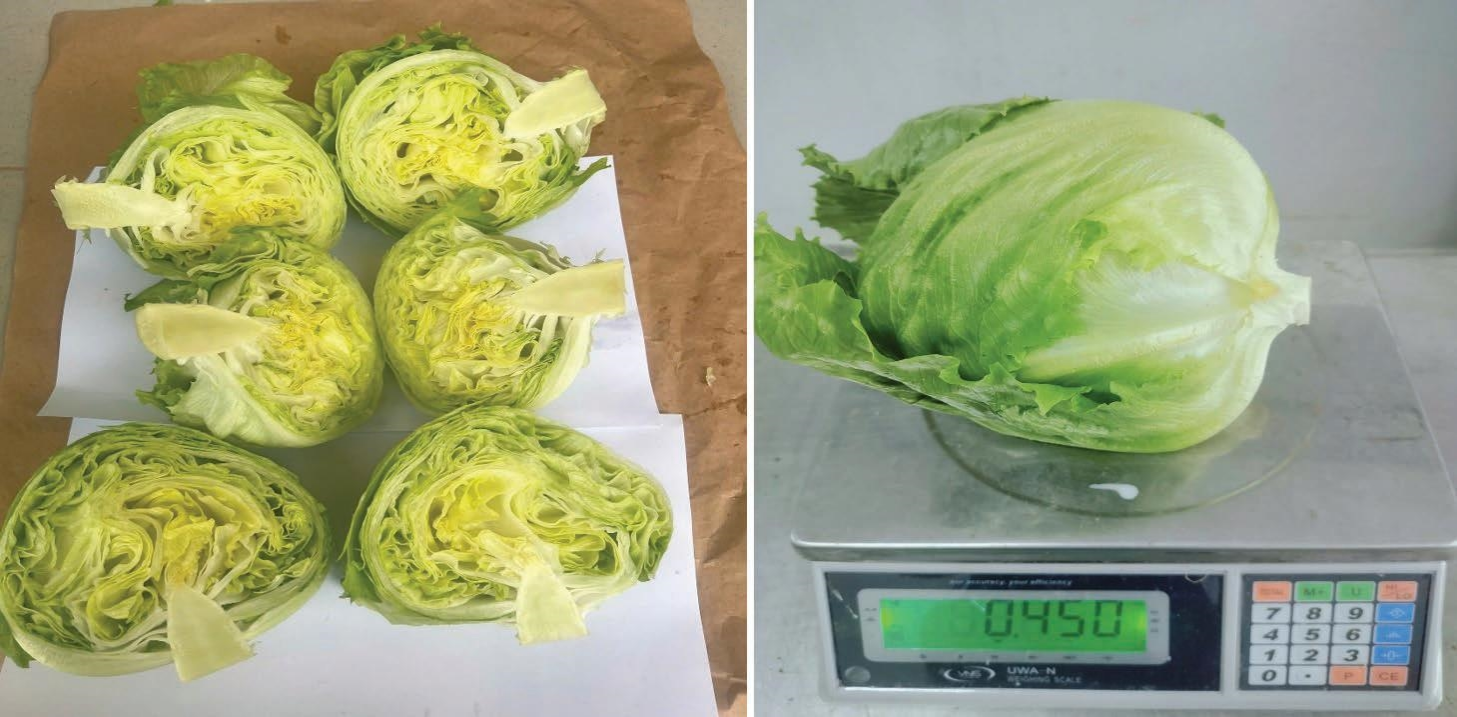
Image of iceberg lettuce.

Iceberg lettuce (*Lactuca sativa* var. *capitata*) variety “Saula” (produced by Enza company) was obtained from Lac Duong farm (belonging to Wineco company), Lam Dong Province, Vietnam. The image of iceberg lettuce is shown in Figure 1. The dimensions and physical properties of the packaging materials and films are presented in Table 1.

**Table 1:** Dimensions and physical properties of the packaging materials and films.

Order	Packaging materials	Length (cm)	Width (cm)	Height (cm)	Thickness	Number of holes and diameter
1	Carton box	52.4	38.9	42.0	0.4 cm	4 holes with 2.0 cm diameter and 2 holes for carrying
2	Carton box glued with LDPE film	52.5	33.5	26.5	0.4 cm	4 holes with 2.0 cm diameter and 2 holes for carrying
3	Danpla plastic box	39.8	31.7	20.0	0.45 cm	4 holes with 2.25 cm diameter and 2 holes for carrying
4	Green MAP bag	48.5	30	—	20 μm	Nonperforated
5	LDPE bag	—	—	—	15 μm	Nonperforated

*Note:* The thicknesses of the packaging materials and film were measured using a digital caliper and micrometer (ID C112, Mitutoyo, Japan).

#### 2.1.2. Packaging Materials

**Figure 2:**
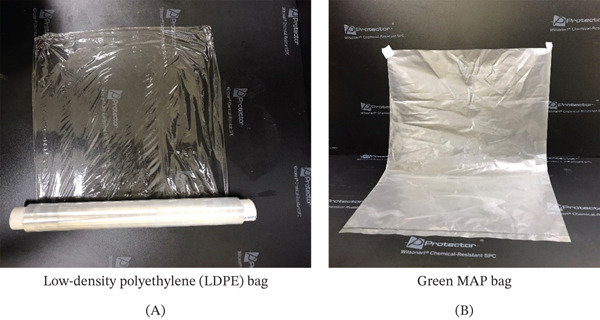
Primary packaging.

**Figure 3:**
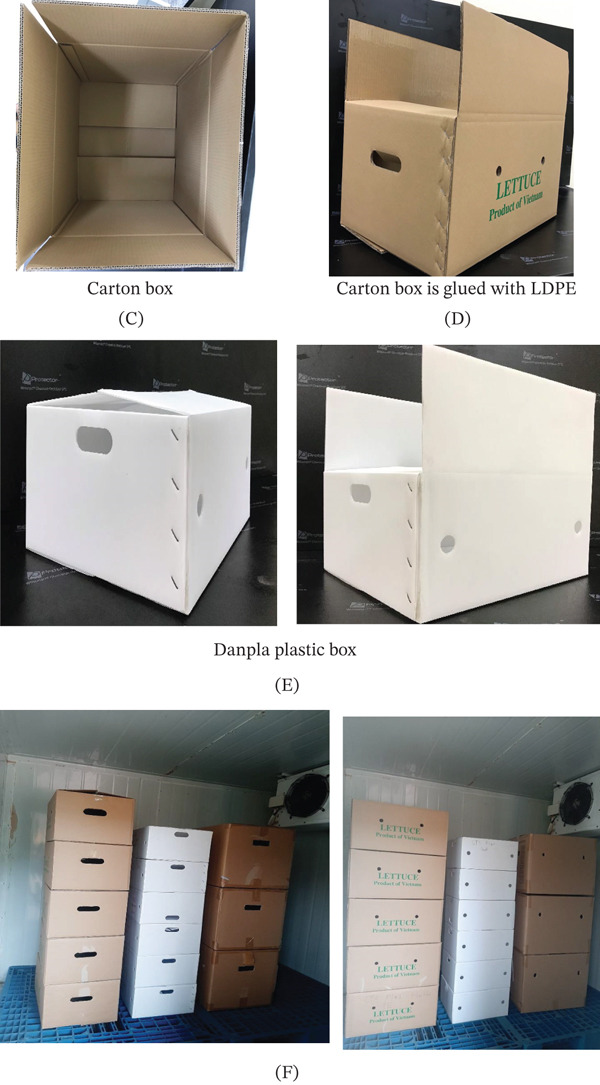
Secondary packaging materials and their arrangement in the cooling chamber.

Primary and secondary packaging materials were produced by the IVINA packaging company of Vietnam. Therefore, the primary packaging materials (Figure 2) included low‐density polyethylene (LDPE) and green MAP, whereas the secondary packaging materials (Figure 3) consisted of a carton box, a carton box glued with a layer of LDPE film, and a Danpla plastic box. Plastic pallets were also purchased for carrying and facilitating handling. These materials were selected for commercial use, particularly for exportation purpose. For each combination of primary and secondary packaging, 12 heads of iceberg lettuce were packed for subsequent evaluation of chemical quality parameters. The types of primary packaging used are illustrated in Figure 2.

### 2.2. Research Methods

#### 2.2.1. Experimental Designs

##### 2.2.1.1. Determine the Optimum Packaging Mode for Iceberg Lettuce

The experiment was conducted with a combination of two factors: primary and secondary packaging materials. Lettuces were harvested early in the morning at the optimal maturity stage of 58 days after planting [6]. After harvesting, the samples were immediately wrapped in LDPE, packed in carton boxes, and transported at night by cold truck to the Postharvest Technology Laboratory. After that, samples were manually cleaned using tissue, and damaged leaves were manually removed. Thereafter, cleaned samples that were free from any damages and defects were kept in a cool chamber storage at 3^°^C ± 2^°^C and 95% relative humidity. Quality parameters such as TSSs, vitamin C, phenolics, total chlorophylls (total Chls), and carotenoids were evaluated at 6‐day intervals. The experiment was arranged using nine treatment combinations, as shown in Table 2.

**Table 2:** The primary and secondary materials combination.

Treatment combinations	Primary packaging materials	Secondary packaging materials
1	Without primary packaging	Carton box
2	Without primary packaging	Carton box is glued with LDPE film
3	Without primary packaging	Danpla plastic box
4	Wrapped by LDPE	Carton box
5	Wrapped by LDPE	Carton box is glued with LDPE film
6	Wrapped by LDPE	Danpla plastic box
7	Wrapped by Green MAP	Carton box
8	Wrapped by Green MAP	Carton box is glued with LDPE film
9	Wrapped by Green MAP	Danpla plastic box

The packaged iceberg lettuce experiments were stored at 3^°^C ± 2^°^C and 95% relative humidity. All boxes were placed on the plastic pallets as shown in Figure 3D. The experiment was conducted in a completely randomized design (CRD) with nine treatment combinations, three replications, and 27 total experimental units.

The following parameters were determined every 6 days until the end of storage, and samples were selected randomly:

1. TSS (°Brix)
2. Vitamin C (mg/100 g)
3. Phenolics (mg GAE/g FW)
4. Total Chl (mg/g)
5. Carotenoid (mg/g)

### 2.3. Analytical Methods

#### 2.3.1. TSS

TSSs were determined using a digital refractometer (PAL‐1, LRO3^*^2, Tokyo, Japan) at room temperature [16, 17]. The results are expressed as degree Brix (°Brix).

#### 2.3.2. Vitamin C

Vitamin C was determined by redox titration using iodine solution in the presence of potassium iodide [18, 19] by using Titroni basic (D‐55122 Mainz, Berlin, Germany). Five (5) grams of the sample was extracted in 5 mL of 5% HCL using a pestle and mortar. The extracted sample was poured into a 50‐mL volumetric flask, diluted with distilled water, stored in the dark for 10 min, and filtered using filter paper. The clean filtered sample solution was used to determine vitamin C through titration directly with 0.01 N of iodine solution until a blue color was formed. One percent starch was used as an indicator and a distinct rose‐pink endpoint lasting for 15 s.

The vitamin C content was estimated using the equation below:

Vitamin C%mg/g=0.00081000100∗a∗V∗∗v.c,

where

*a* = number of milliliters (mL) of I_2_ used for titration.

*V* = total volume of extract (50 mL).

*c* = weight of materials to be analyzed (grams).

*v* = number of milliliters (mL) of sample solution to be analyzed (10 mL).

0.00088 = grams of vitamin C equivalent to 1 mL 0.01 N.

#### 2.3.3. Total Phenolic Content (TPC)

The TPC was determined by Folin–Ciocalteu reagent (FCR) using gallic acid as A standard [20]. Iceberg lettuce (5 g) was extracted three times with 25 mL of 99.8% methanol by grinding using a mortar and pestle. Then, the extract was homogenized using a homogenizer (IKA, T25 digital ULTRA‐TURRAX), waited for 30 min at room temperature in a dark place, and centrifuged for 10 min at 7000 rpm at 4°C (Centrifuge 5810R, Eppendorf, Hamburg, Germany). Gallic acid was used as the standard for the calibration curve. A stock solution (1 mg/mL) was prepared by dissolving 0.1 g of gallic acid in 100 mL of distilled water. Various concentrations of gallic acid solutions (0, 10, 20, 30, 40, and 50 *μ*g/mL) were prepared.

A blank solution was prepared from all reagents except the gallic acid standard and the sample extract. After extraction, 0.5 mL aliquots of the supernatant were transferred to test tubes and 10% (1:10 dilution with distilled water) 0.2 N FCR (2.5 mL). Finally, after 5 min of FCR addition, 7.5% Na_2_CO_3_ (2 mL) was added to the solution to make a final volume of 5 mL and vortexed to mix well. After 2 h in a dark storage place, the absorbance was measured using a UV spectrophotometer (UV‐1900i, Shimadzu Kyoto, Japan) at a wavelength of 765 nm. The TPC of the extracts was expressed as milligrams of gallic acid equivalents (mg GAE) per gram of fresh sample and calculated using the following formula:

TPC mg GAE/g FW=c∗vm,

where TPC is the TPC milligrams of gallic acid equivalents per gram (mg GAE/g) FW, “*c*” is the concentration of gallic acid from the calibration curve in milligrams per milliliter (mg/mL), “*v*” is the volume of extract in milliliters (mL), and *m* is the mass of sample in grams (g).

#### 2.3.4. Total Chl and Carotenoids

Chlorophyll content was determined in terms of total Chl from chlorophyll a (Chl a) and chlorophyll b (Chl b) submissions. Chl a, Chl b, and carotenoid contents were determined according to the method described by Jamie and Saltveit [21]. Five grams (5 g) of the upper portion of an iceberg lettuce sample, closer to the leaf apex, was weighed and ground using a mortar and pestle in 20 mL of 80% acetone (acetone and water were mixed in an 80:20 ratio). The mixture was then homogenized (homogenizer, IKA, T25 digital ULTRA‐TURRAX), placed in the dark for 30 min at room temperature, and centrifuged (cold centrifuge 5810R, Eppendorf, Hamburg, Germany) at 7000 rpm at 4°C for 10 min. The homogenate was filtered through four layers of cotton cloth. Then, the supernatant was carefully transferred to a volumetric flask, and the volume was increased to 25 mL with 80% acetone. The supernatant was then used for measuring Chl a, Chl b, and total carotenoids at 662, 646, and 470 nm absorbance with a UV–VIS recording spectrophotometer (UV‐1900i, Shimadzu Kyoto, Japan). Chl a and Chl b contents were calculated using the equation below.

Then, absorbance (*A*) is read from the UV spectrophotometer.

Chl a mg/g FW=12.72.69 A662− A646∗V1000∗W,Chl b mg/g FW=22.94.68 A646− A662∗V1000∗W,Total Chl mg/FW=Chl a mg/g FW+Chl b mg/g FWtotal carotenoid mg/gFW=10003.270104 A470− Chl a− ∗V1000 W,

where *V* is the volume of supernatant (mL); *A* is the absorption at wavelengths 662, 646, and 470 nm; and *W* is the weight of the sample (g).

The data were expressed as milligrams per gram (mg/g) FW total Chl and carotenoid as milligrams per gram (mg/g) FW.

### 2.4. Statistical Analysis

Data were analyzed by analysis of variance (ANOVA) using Minitab 16 statistical software (LLC, Pennsylvania, United States) and MS Excel software. Prior to analysis, assumptions of homogeneity of variance and normality were tested. Data comparison was performed using Fisher′s multiple comparison tests to determine the significance of differences among treatments at a 95% confidence level. Each value was determined three times. Results are given as mean ± SD (standard deviation).

## 3. Results and Discussions

### 3.1. Effect of Packaging on the TSS

**Figure 4:**
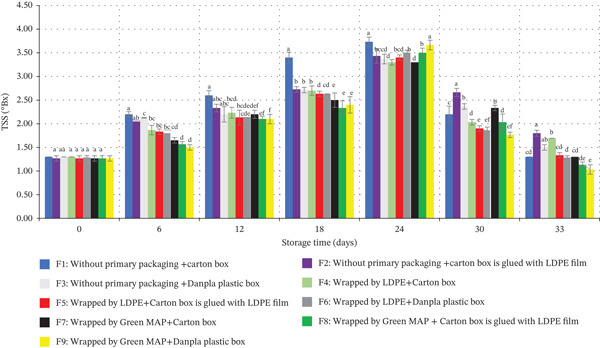
Effect of packaging on the total soluble solids of iceberg lettuce during cold storage. *Note:* Vertical bars represent the standard deviation of the mean of three replications; bars with different letters indicate significant differences (*p* < 0.05) according to Fisher′s comparison test.

The effect of packaging materials on the TSS of iceberg lettuce during cooling chamber storage at 3^°^C ± 2^°^C and 95% relative humidity is presented in Figure 4. According to the results (Figure 4), the TSS content ranged from 1.2°Brix to 3.77°Brix. The TSS values gradually increased up to 24 days. The increase in the TSS parameter during storage could be potentially caused by maturation and moisture loss as a result of the concentration of juice formed. The work by Schvambach et al. [22] found that the TSS of lettuce increased from 2.82°Brix to 3.3°Brix during storage time for crisp lettuce. The difference might be due to variety, maturity stage, packaging material properties, and storage conditions.

The samples without primary packaging in carton boxes showed significantly (*p* = 0.05) the highest TSS values at 12, 18, and 30 storage days, which could be mainly due to moisture loss, the formation of concentrated juice, and anticipating maturation. Scholars also reported that fruits packaged in open plastic crates were observed to exhibit lower firmness and higher TSS levels due to water loss, as pectin structural matrices are changing into soluble materials and increased transpiration rates resulted in faster softening tissue and decaying [23]. However, after 24 storage days, it started to decrease due to the consumption of TSSs used as substrate for respiration. Indeed, when a horticultural product is harvested at the optimum point of maturation, the sugar is used as a substrate during respiration. Similar trends were reported by Brizzolara et al. [24], who observed the preservation of TSS in crisp lettuce under different postharvest storage conditions.

The results (Figure 4) showed that during early storage, the lowest TSS was recorded for samples wrapped by LDPE and green MAP, particularly for samples wrapped by green MAP and packed in Danpla. This is due to the fact that this packaging method reduced water loss and maintained a humid microclimate inside these films, which delayed ripening and resulted in slow hydrolysis of starch, hemicellulose, pectic acid, and proteins, thus lowering the TSS.

The previous works showed that the reduction in water loss in the iceberg lettuce, which prevented the development of juice concentration and decreased respiratory metabolism by increasing carbon dioxide and decreasing oxygen within the packages, is most likely caused by the permeability of these packing materials to water vapor. The different changes in the TSS can be attributed to the slower respiration rate as well as the metabolic activities, which slow down the ripening process in packaging materials [25].

However, after 24 storage days, it starts to decline as the cell membrane starts to degrade and starch is lost. The lower TSS at the end of storage time also indicates a lower sugar level, which again limits the sweetness of the iceberg lettuce and affects consumer desire. During 30–33 days of evaluation storage time, samples without primary packaging packed in a carton box glued with LDPE film are the best mode in preservation of TSSs. This might be because these packaging materials prevent cell membrane damage, reduce transpiration, and preserve starch by decreasing carbohydrate consumption due to respiration. It also might be due to maintaining the quality of postharvest whole lettuce by protecting the mitochondrial structure and turgor pressure and reducing transpiration, enzymatic, and microorganism activities. The work by Iakimova et al. [26] found that tomato fruit ripening conditions support this work.

### 3.2. Effect of Packaging on Vitamin C Levels

**Figure 5:**
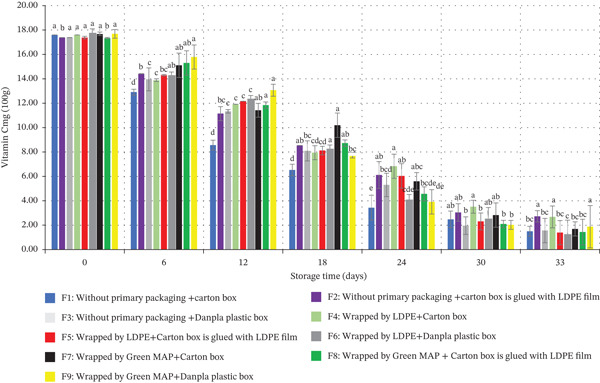
Effect of packaging materials on the vitamin C content of iceberg lettuce during cold storage. *Note:* Vertical bars represent the standard deviation of the mean of three replications; bars with different letters indicate significant differences (*p* < 0.05) according to Fisher′s comparison test.

The effect of different packaging materials on the vitamin C content of iceberg lettuce during storage in a cooling chamber at 3^°^ *C* ± 2^°^ *C* and 95% relative humidity is presented in Figure 5. The results showed a gradual decrease in vitamin C content across all packaging materials over the storage period of more than 1 month. The highest vitamin C content, measured on Day 0, was 17.6 mg/100 g. The greatest loss of vitamin C (1.4 mg/100 g) was observed after 33 days in samples without primary packaging, which were packed only inside carton boxes. The reduction in vitamin C during storage is attributed to the chemical oxidation of ascorbic acid to dehydroascorbic acid. This degradation may also be promoted by oxidative processes stimulated by light, oxygen, temperature, and enzymatic activity, including ascorbate oxidase and peroxidases, as well as other environmental factors [27].

The previous reports by Selma et al. [28] for romaine lettuce showed that the decreased vitamin C concentration was due to the increased peroxidase activity in the lettuce leaf during storage. A report by Robertson [29] also implied that the loss may be due to the inability of the packaging materials to act as an effective barrier against light, oxygen, temperature, and other environmental factors. The reduction of vitamin C during storage time also agrees with the work by Mahajan et al. [30], who studied the effect of packaging and storage environments on the quality and shelf life of bell pepper. The present result is supported by an earlier report by Patil et al. [31], who found the postharvest behavior of different lettuce cultivars and their cut form under different storage conditions. The result at 6 and 12 days of storage with samples wrapped in green MAP and packed in Danpla plastic boxes exhibited the highest vitamin C levels. This might be due to packaging materials lowering oxidation rates and reducing moisture loss by increasing relative humidity inside the packaging and preservation. According to Paneru [32], the increasing trend in the ascorbic acid level may be caused by the cell wall breaking down as ripening progresses, supplying substrates for the synthesis of ascorbic acid. The influence of packaging films on maintaining a higher ascorbic acid content in okra has also been reported [33].

On the 18^th^ day of evaluation, samples wrapped with green MAP and packed in carton boxes exhibited the highest vitamin C content. This might be due to the provision of a modified atmosphere that is used for the reduction of oxidation and water loss. This agrees with the observation of Fadeyibi [34], who found that wrapping fruits with plastic film to reduce water loss helped retain ascorbic acid even more than the optimal storage temperature. However, the vitamin C content of lettuce wrapped in MAP started to decline relatively faster after 12 days of storage when compared with other treatments. This could be because of condensation and increased respiration heat, which makes the vitamin thermally unstable inside the packaging material. Additionally, the package′s extremely low permeability limits air passage from the inside to the outside of the packaging materials, which leads to heat buildup and a decrease in vitamin C concentration. Higher CO_2_ formed and may stimulate the oxidation of ascorbic acid, probably due to the activation of ascorbate peroxidase, as a result reducing vitamin C [35].

However, after 3 weeks of storage, samples wrapped in LDPE and packed in a carton box and samples without primary packaging and packed in a carton box glued with LDPE preserved vitamin C content better than the rest of the treatments (Figure 5). At 33 days, samples without primary packaging and packed in carton boxes glued with LDPE film and samples wrapped with LDPE and packed in carton boxes exhibited the highest vitamin C content. This might be because these materials maintained the appropriate respiration intensity, modified the atmosphere, and effectively inhibited the growth of microorganisms. The work of Xu et al. [36], who found that modified atmosphere packaging (MAP) preserved fresh‐cut iceberg lettuce, supports this report.

### 3.3. Effect of Packaging on the Phenolic Content

**Figure 6:**
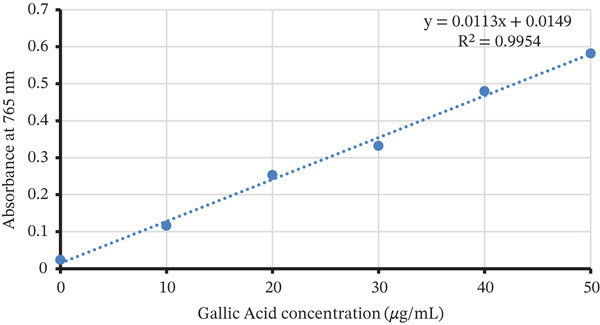
Gallic acid calibration curve.

**Figure 7:**
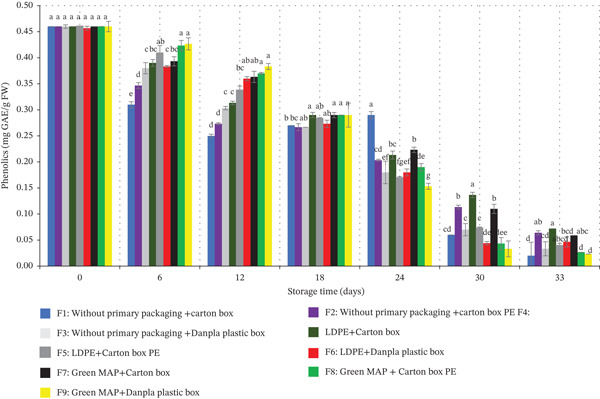
Effect of packaging on the phenolic content of iceberg lettuce during cold storage. *Note:* Vertical bars represent the standard deviation of the mean of three replications; bars with different letters indicate significant differences (*p* < 0.05) according to Fisher′s comparison test.

The result of TPC were derived from a calibration curve equation (*y* = 0.0113*x* + 0.0149, *R* ^2^ = 0.9954) of gallic acid concentration (0–50 *μ*g/mL) Figure 6 and Table 3 and expressed in milligram gallic acid equivalents (mg GAE) per gram of extract. The effect of different packaging materials on the TPC of iceberg lettuce during cooling chamber storage at 3^°^C ± 2^°^C and 95% relative humidity is presented in Figure 7.

**Table 3:** Calibration data for the gallic acid standard curve (0–50 *μ*g/mL; *y* = 0.0113*x* + 0.0149, *R* ^2^ = 0.9954), expressed as mg GAE/g extract.

Concentration (mcg/mL)	Absorbance 765 nm
0	0.024
10	0.116
20	0.253
30	0.332
40	0.48
50	0.582

*Note:* Instruments, chemicals, and reagents used: Titroni basic (D‐55122 Mainz, Berlin, Germany), cold centrifuge (Centrifuge 5810R, Eppendorf, Hamburg, Germany), homogenizer or shaker (IKA, T25 digital ULTRA‐TURRAX), electronic refractometer (PAL‐1, LRO3*2, Tokyo, Japan), and UV spectrophotometer (UV‐1900i, Shimadzu Kyoto, Japan). Gallic acid (Xilong Chemical Co. Ltd., Shantu, China), 2 N Folin–Ciocalteu′s reagent (FCR) (Sigma Aldrich, India), HCL99% (Warsaw, Poland), 80% acetone and sodium carbonate (Xilong Chemical Co. Ltd., Shantu, China), iodine (Tokyo, Japan), starch (Shantu, China), and potassium iodide and 99.8% methanol (Xilong Chemical Co. Ltd, Shantu, China) were purchased. Standard solutions were prepared according to the procedures used.

The highest phenolics (0.46 mg GAE/g FW) were recorded on 0 days of storage time, and statistically, there was no significant difference among the packaging methods (*p* = 0.38). The result (Figure 7) shows that the total phenolic compounds decreased across all storage times except for samples without primary packaging, packed inside the carton box at 18 and 24 storage days. The reduction could be due to reduced plant biochemical processes and enzymatic activities, such as polyphenol oxidase and peroxidase enzymes. The work reported by Viacava et al. [37] showed that the loss of polyphenols during the storage of lettuce may be related to the enzymatic oxidation of phenolic compounds by polyphenol oxidase and peroxidase. Across the storage periods, samples showed a slight reduction of total phenolic compounds, which may be due to the use of polyphenols as substrates for the polyphenol enzyme and the conversion between free and bound phenolic substances [38, 39].

Liu et al. and Jirakitticharoen et al. [40, 41] found the decrease of phenolic content in lamb lettuce (*Valerianella locusta* [L.] Laterr) and romaine lettuce (*Lactuca sativa* L. var. *longifolia*) during storage under MAP at 8°C.

After 0 days of storage time, the concentration of phenolic content started to decline, particularly for samples without primary packaging in carton boxes. This might be due to the presence of more reactive oxygen, and the cell wall–bound enzymes that facilitate higher oxidation might be released because of water loss and cell membrane tissue damage. The decrease in the total phenolics results from the oxidation and hydrolytic activities of phenolic compound enzymes in the presence of more oxygen on iceberg lettuce [42].

However, the phenolic concentration of samples without primary packaging, packed in carton boxes, increased during cooling chamber storage times of 18 and 24 days. The wilting, water loss, and tissue breakdown of the leaves may be the cause of the increased phenolic concentration, which is linked to a decrease in the activity of the polyphenol oxidase enzyme. It also might be because cold storage induces the accumulation of phenolic content, and lettuce induces antioxidant production as a defense mechanism against stress. The work by Lịchanporn et al. [43] and Mustafa et al. [44] revealed that the phenylalanine ammonia lyase enzyme may contribute to an increase in total phenolic compounds. Similarly, Mai and Glomb [45] also reported that phenylalanine ammonia lyase activity could have increased in response to microbial decay and mechanical damage and could have resulted in an increased biosynthesis of phenolics during storage. The work conducted by Rivera et al. [46], who found effects of ascorbic acid applied by two hydrocooling methods on the physical and chemical properties of green leaf lettuce stored at 5°C, also supports this work.

According to the results (Figure 7), during early storage time, samples wrapped with green MAP and LDPE preserved phenolic concentration, especially samples wrapped with green MAP and packed in Danpla plastic boxes. This might have occurred because these packaging materials prevent moisture loss and oxidation. However, the reduction of TPCs after 18 days of storage in these packaging materials might be due to an excessive accumulation of CO_2_ concentration in these packaging materials, which prevents the activity of the phenylalanine ammonia lyase enzyme. Packaging materials could have increased internal CO content accumulation, triggering anaerobic respiration, which in turn led to the production of free radicals and membrane damage. As a result of this process, an increased accumulation of CO_2_ might induce the polyphenol oxidase enzyme activity, which possibly accelerates the oxidation of polyphenols. This result agrees with the work of Venkatachalam [47], who found internal browning disorder in Chinese cabbage (*Brassica rapa* L. ssp. *chinensis*) and Pacific rose apples. Luna et al. [48] reported that a high CO_2_‐modified atmosphere significantly inhibits phenolic accumulation in fresh‐cut lettuce and carrots due to its ability to inhibit phenylalanine ammonia lyase.

The metabolic conversion of the TPC into secondary phenolic compounds and the increase in humidity caused by the building of water vapor, which resulted in the generation of heat and damage to the cell membranes of the samples, could be the cause of the decrease in the TPC. The work of Paulus et al. [49], in which the lowest total phenolic losses in okra fruits packed in LDPE of 30 and 40 *μ*m thickness after 28 days of storage were 9.67% and 11.19%, respectively, also supports this work.

The result showed that, after 24 days of storage, iceberg lettuce wrapped with LDPE and green MAP film inside the carton box container and samples without primary packaging put in the carton box glued with LDPE showed higher TPC. During evaluation periods of 30 and 33 days of storage time, samples wrapped by LDPE and packed inside the carton box exhibited the highest TPC. This might be due to the permeability properties of packaging materials, which preserve samples by regulating and controlling the gas composition and proportion of packaging materials to maintain a lower metabolic process rate, low consumption of respiratory substrates, and lower anaerobic respiration, thus delaying the maturity of fresh samples. This might also be due to the permeability of these packaging materials, creating a desirable atmosphere around the samples and maintaining qualitative characteristics such as phenolics. The work of [50, 51], who found that preservation of phenolic compounds in pomegranate arils packaged with polyethylene and polyester may be due to the increase in the phenylpropanoid pathway under low oxygen stress, agrees with this work.

### 3.4. Effect of Packaging on Total Chl and Carotenoid Content

#### 3.4.1. Total Chl Content

**Figure 8:**
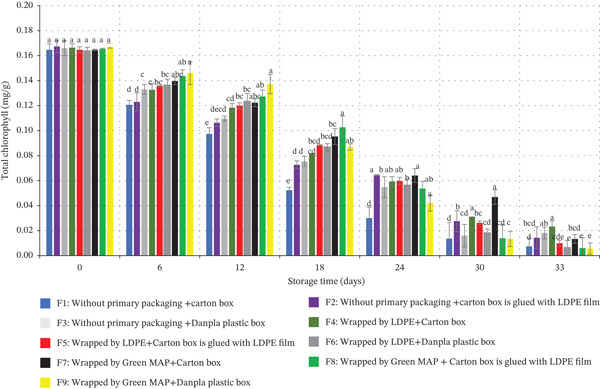
Effect of packaging on the total chlorophyll content of iceberg lettuce during cold storage. *Note:* Vertical bars represent the standard deviation of the mean of three replications; bars with different letters indicate significant differences (*p* < 0.05) according to Fisher′s comparison test.

Chlorophyll is considered to be responsible for the green color, and changes in chlorophyll content can act as a good indicator of leaf senescence during storage. The work by Manolopoulou and Varzakas [52] implied that chlorophyll is the primary source of green color in leafy vegetables and is the most important criterion for consumer perception, and its degradation is the first visible symptom of aging. The present results (Figure 8) show the effect of packaging materials on the total Chl content of iceberg lettuce during cooling chamber storage of 3^°^C ± 2^°^C and 95% relative humidity. The chlorophyll content decreased gradually throughout the storage period. The highest (0.17 mg/g FW) chlorophyll concentration was recorded on 0 days of storage time, and there was no statistically significant difference among packaging methods (*p* > 0.05).

The reduction of chlorophylls across storage time agrees with the work of [15] on fresh‐cut iceberg lettuce, Zhan et al. [53] on tissue browning and antioxidant properties of fresh‐cut baby leaf lettuce, and Lee and Chandra [54] on postharvest qualities of leaf lettuce during packaging. The work by Islam et al. [39] also showed that chlorophyll degradation is associated with leaf yellowing in harvested leafy vegetables, and the decrease might be due to the change from chlorophyll concentration to anthocyanin concentration.

The lowest chlorophyll concentration across storage time was shown by samples that had no primary packaging and were packaged in carton boxes and were significantly affected (*p* < 0.05). This was due to moisture loss, higher oxidation, environmental factors like light, and degradation of chlorophyll by chlorophyllase enzyme. Because senescence and lipid peroxidation in cell membranes are associated with chlorophyll decomposition, the chlorophyll content was lowered. Therefore, the loss of chlorophyll is primarily due to senescence associated with the loss of membrane lipids and proteins, leading to textural changes and cell death. Paulsen et al. [55] found that the effect of MAP on the quality and bioactive compounds of Chinese cabbage supports this finding.

According to the present results, the samples wrapped with LDPE and green MAP film inside Danpla plastic boxes and carton boxes glued with LDPE revealed higher concentrations of chlorophyll during early storage. This might be due to lower permeability and higher thickness, preventing moisture loss, lowering the effect of environmental factors, and inhibiting enzymatic oxidation as a result. It is also due to the fact that the possible reason for maintaining those quality traits could be the consequences of accumulated CO_2_ preventing chlorophyll loss and depleting oxygen concentration in the inside atmosphere of packages. Elevated CO_2_ and lower concentrations of oxygen can retard yellowing, chlorophyll, and protein degradation, which improves the overall acceptance of leafy vegetables [56–58].

However, after 2 weeks of storage, the chlorophyll concentration in these packaging materials declined dramatically, especially for samples wrapped in green MAP film inside the Danpla plastic box. This might be because high concentrations of CO_2_ can cause cell membrane damage and lead to physiological disorders. The report by Tudela et al. [59] showed that the elevated CO_2_ concentration and lower O_2_ concentration observed in the oriented polypropylene packaging with 0.3% oxygen and 25% CO_2_ could have induced cytoplasmic acidification and affected mitochondrial function, which could have resulted in oxidative damage in leaf tissue. Therefore, the observed decrease in chlorophyll content in iceberg lettuce wrapped with LDPE and green MAP film inside the Danpla plastic box and carton box glued with LDPE could be linked to cell membrane damage and loss of cell membrane integrity. In addition, similar observations were also reported in lettuce leaves by [60].

The result (Figure 8) showed that after storage for 24 and 30 days, samples wrapped in LDPE and green MAP films and packed inside the carton box, as well as samples without primary packaging inside the carton box glued with LDPE, showed higher concentrations of chlorophylls. At 30 days of evaluation, samples wrapped with green MAP and packed inside the carton box significantly (*p* = 0.00) showed the highest chlorophyll concentrations. This might be because these packaging materials had a high protective ability to retain and preserve chlorophyll in iceberg lettuce for an improvement in shelf life. At 33 days of storage period, samples wrapped in LDPE and packed in a carton box exhibited the highest values of chlorophylls significantly (*p* = 0.01) and showed the highest values, but statistically there was no significant difference in samples with no primary packaging packed in a Danpla plastic box. This may be because it protects the membrane from damage and regulates environmental factors like light. The accumulating CO_2_ and decreased O_2_ content in the packages′ inside atmosphere may also contribute to the maintenance of certain quality attributes. In agreement with this result, similar chlorophyll preservation trends by MAP have been reported by other studies on the preservation of fresh‐cut iceberg lettuce [61, 62].

#### 3.4.2. Total Carotenoid Content

**Figure 9:**
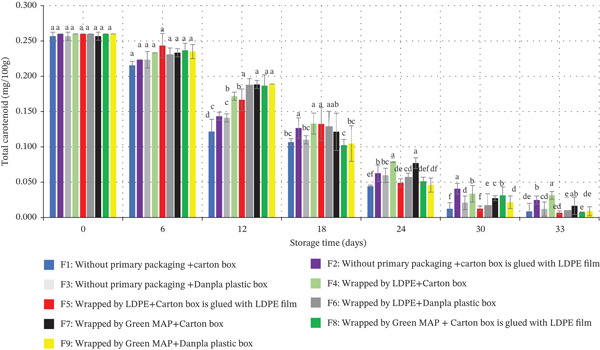
Effect of packaging on the total carotenoid content of iceberg lettuce during cold storage. *Note:* Vertical bars represent the standard deviation of the mean of three replications; bars with different letters indicate significant differences (*p* < 0.05) according to Fisher′s comparison test.

The effect of packaging materials on the total carotenoid content of iceberg lettuce during cooling chamber storage at 3^°^C ± 2^°^C and 95% relative humidity is presented in Figure 9. According to our findings, carotenoids decreased for all packaging methods. The highest carotenoid content (0.26 mg/g FW) was recorded at 0 days of storage time, and there was no significant difference for 0 and 6 days of storage periods (*p* > 0.05), while carotenoid content decreased across all storage times. The decreased carotenoid content of iceberg lettuce during storage may be attributed to environmental factors such as light, temperature, moisture loss, heat formation, oxidation, and activity of the lipoxygenase enzyme, which may degrade carotenoid pigment [63, 64]. Oxidation of carotenoids is caused by exposure to oxygen and is facilitated by lipoxygenase, an enzyme that also oxidizes lipid molecules. Low moisture content during vegetable processing may also result in concentration losses of carotenoids. The reduction of carotenoids across storage time for crisp lettuce in different postharvest storage conditions was observed [65]. During storage time, samples with no primary packaging in a carton box exhibited the highest loss of carotenoids, which might be due to environmental factors that could hasten pigment deterioration, loss of water, and oxidation, leading to reduced chlorophylls and, as a result, reduced carotenoids. Similar reduction of carotenoids was observed in Chinese cabbage during cold storage [66].

As in the case of chlorophylls, at the early storage time until the 12^th^ day, the iceberg lettuce wrapped in green MAP film and packed inside the Danpla plastic box, packed in a carton box glued with LDPE, and wrapped in green MAP and packed inside a carton box revealed higher concentrations of carotenoid. This might be due to the packaging materials′ properties and thickness, preventing gas exchange and moisture loss as a result of decreased catabolic source reactions like oxidation. However, afterward, carotenoid concentration in these packages started declining due to high condensation formed as a result of heat formation and degradation of the cell membrane of the samples. Additionally, an increase in respiration due to a higher metabolic rate can result in the decay and senescence of samples and, as a result, reduce carotenoid levels. A similar trend was reported by Huang et al. [67], who conducted an experiment on the quality of fresh‐cut broccoli during refrigerated storage. The work by [68] also depicted that high CO_2_ conditions could damage the membrane by stimulating lipid oxidation. MAP with a lower CO_2_ content than 20% helped preserve the chlorophyll content [69, 70]. The authors also reported that anaerobic respiration, caused by a low O_2_ and high CO_2_ environment, limits the quality of vegetables during storage due to the reduction of chlorophylls and carotenoids.

At the 18^th^ day of storage time, samples wrapped in LDPE showed slightly higher carotenoid values, but the difference was not statistically significant (*p* = 0.19). The results during and after 3 weeks of storage showed that samples wrapped with green MAP and LDPE and packed in carton boxes, as well as samples without primary packaging and packed inside carton boxes glued with LDPE film, exhibited higher carotenoid concentration. This might be because these packaging materials preserved the quality of freshness and allowed the exchange of gasses from the interior with the external atmosphere. It also might be due to the suppression of metabolic activity and permeable films, which primarily provide an extended storage life of lettuce. Koukounaras et al. [71] reported that a modified atmosphere is commonly used to prevent the degradation of carotenoids and browning of fresh‐cut lettuce.

## 4. Conclusions

This study intensely revealed that packaging materials significantly affected the chemical quality parameters of iceberg lettuce during cold storage at 3^°^C ± 2^°^C and 95% relative humidity. Samples harvested at 58 DAP and packed in a carton box glued with LDPE film without primary packaging showed the best quality compared with the other packaging materials. This packaging approach preserved TSSs, vitamin C, phenolics, total Chls, and carotenoids. Thus, it can be concluded that iceberg lettuce, the “Saula” variety, packed in a carton box glued with LDPE without primary packaging, could maintain the chemical quality parameters and be used for export over long distances. Based on the limitations of time and resources, it is recommended to conduct further studies on the effect of different storage temperatures and relative humidity using different varieties of lettuce. Future studies should also investigate the effect of packaging on the enzyme′s activity, particularly ascorbate oxidase and polyphenol oxidase, and microorganism growth on the quality of iceberg lettuce during cold storage under different conditions for export purposes.

## Author Contributions

Tolcha Techane Alemu: conceptualization, data curation, analysis, investigation, methodology, validation, visualization, writing original draft, review, and editing. Vu Thi Kim Oanh: writing and drawing of the original paper, supervision, editing, visualization, validation, data curation, conceptualization, methodology, and investigation.

## Funding

This study was funded by the VLIR‐network program approved by the Ministry of Education and Training (No. 1596/QĐ‐BGDĐT) and the VLIRUOS project and the Post‐harvest Technology Department at the Faculty of Food Science and Technology, VNUA, Vietnam, 1.

## Ethics Statement

Review and/or approval by an ethics committee, as well as informed consent, were not required for this study because this article did not involve any direct experimentation/studies on living beings.

## Conflicts of Interest

The authors declare no conflicts of interest.

## Supporting information


**Supporting Information** Additional supporting information can be found online in the Supporting Information section. Table 1: Appearance changes of packaged iceberg lettuce during cold storage (3^°^C ± 2^°^C, 95% RH).

## Data Availability

The data that support the findings of this study are available from the corresponding author upon reasonable request.
