# Supplementary material for: Effect of Packaging Materials on Lettuce (Lactuca sativa var. capitata) Chemical Quality Under Cold Storage
Source: Int J Food Sci. 2026 Jul 2;2026:9968381. doi: 10.1155/ijfo/9968381 (PMC13325404; doi:10.1155/ijfo/9968381)
Supplement: Supplementary file 1 — Supporting Information Additional supporting information can be found online in the Supporting Information section. Table 1: Appearance changes of packaged iceberg lettuce during cold storage (3°C ± 2°C, 95% RH). [file IJFO-2026-9968381-s001.docx]

**Supplementary materials**

**Table 1:** Appearance changes of packaged iceberg lettuce during cold storage (3 ± 2 °C, 95% RH).

| Storage time (Day) | Packaging materials | | | | | | | | |
| --- | --- | --- | --- | --- | --- | --- | --- | --- | --- |
|  | F_1_ | F_2_ | F_3_ | F_4_ | F_5_ | F_6_ | F_7_ | F_8_ | F_9_ |
| 0 | 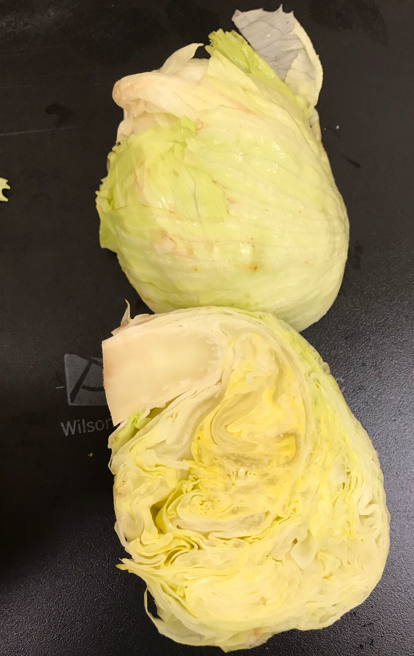 | 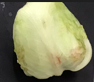  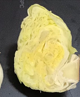 | 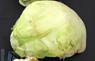  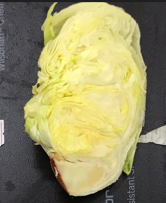 | 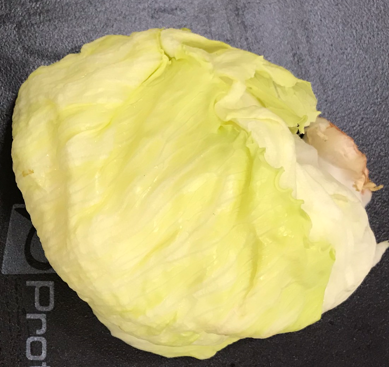  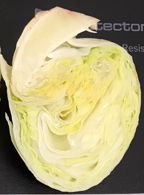 | 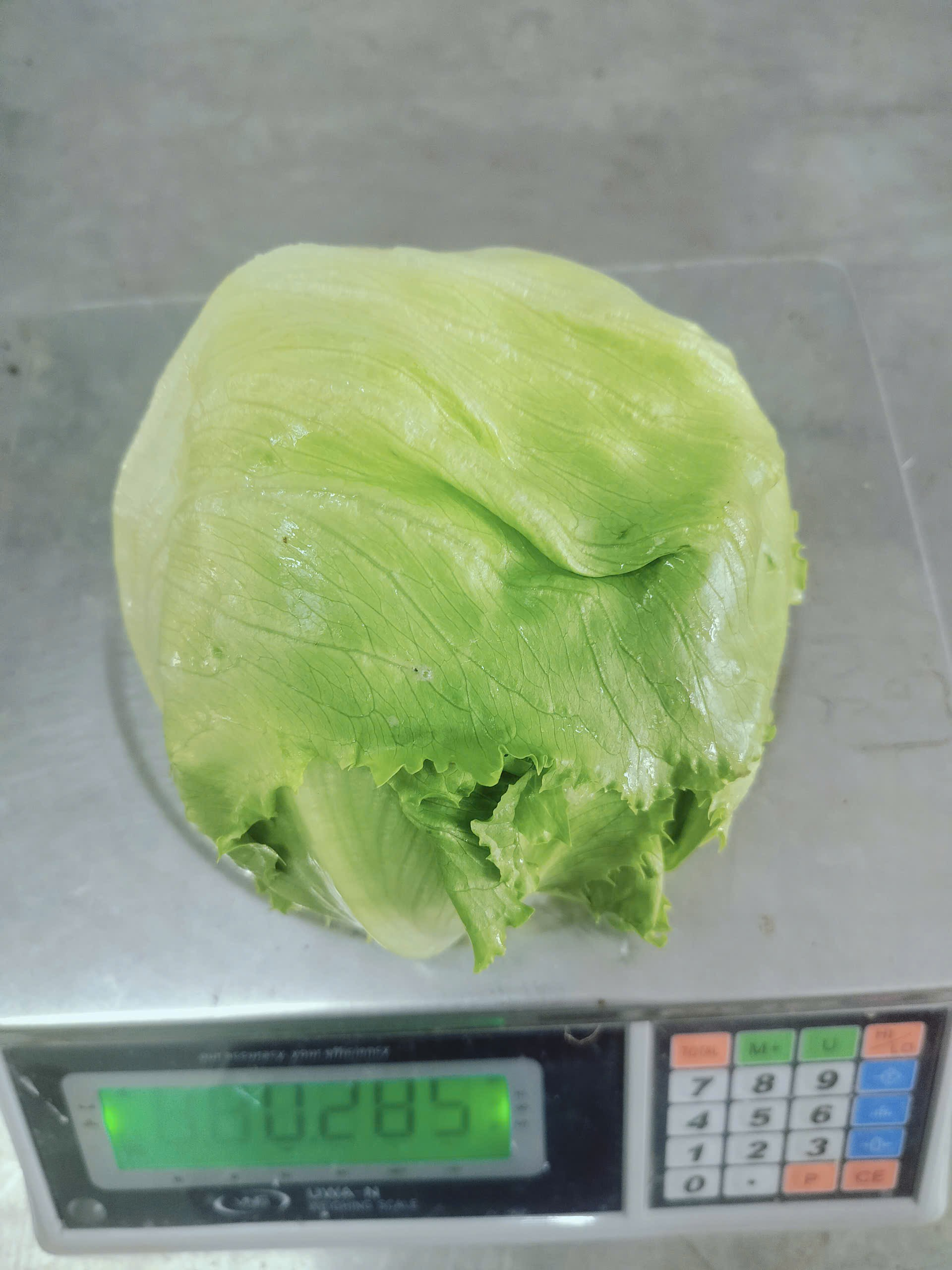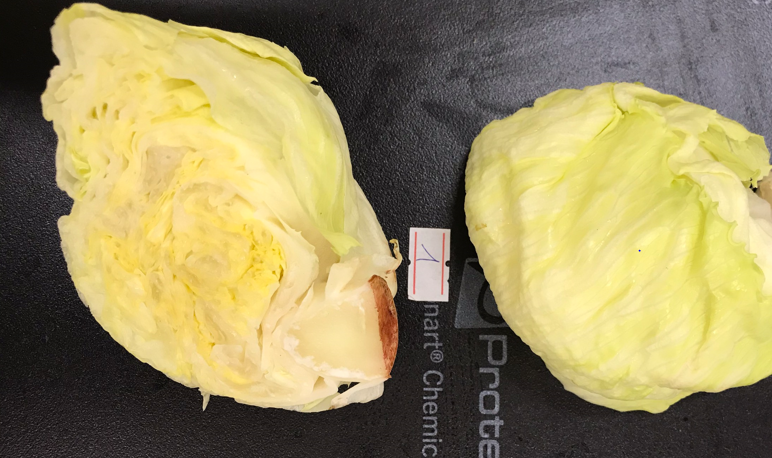 | 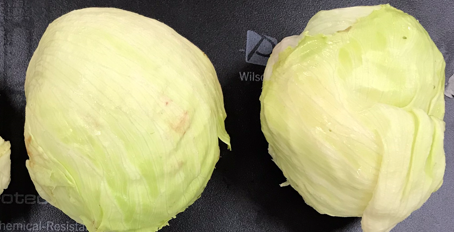  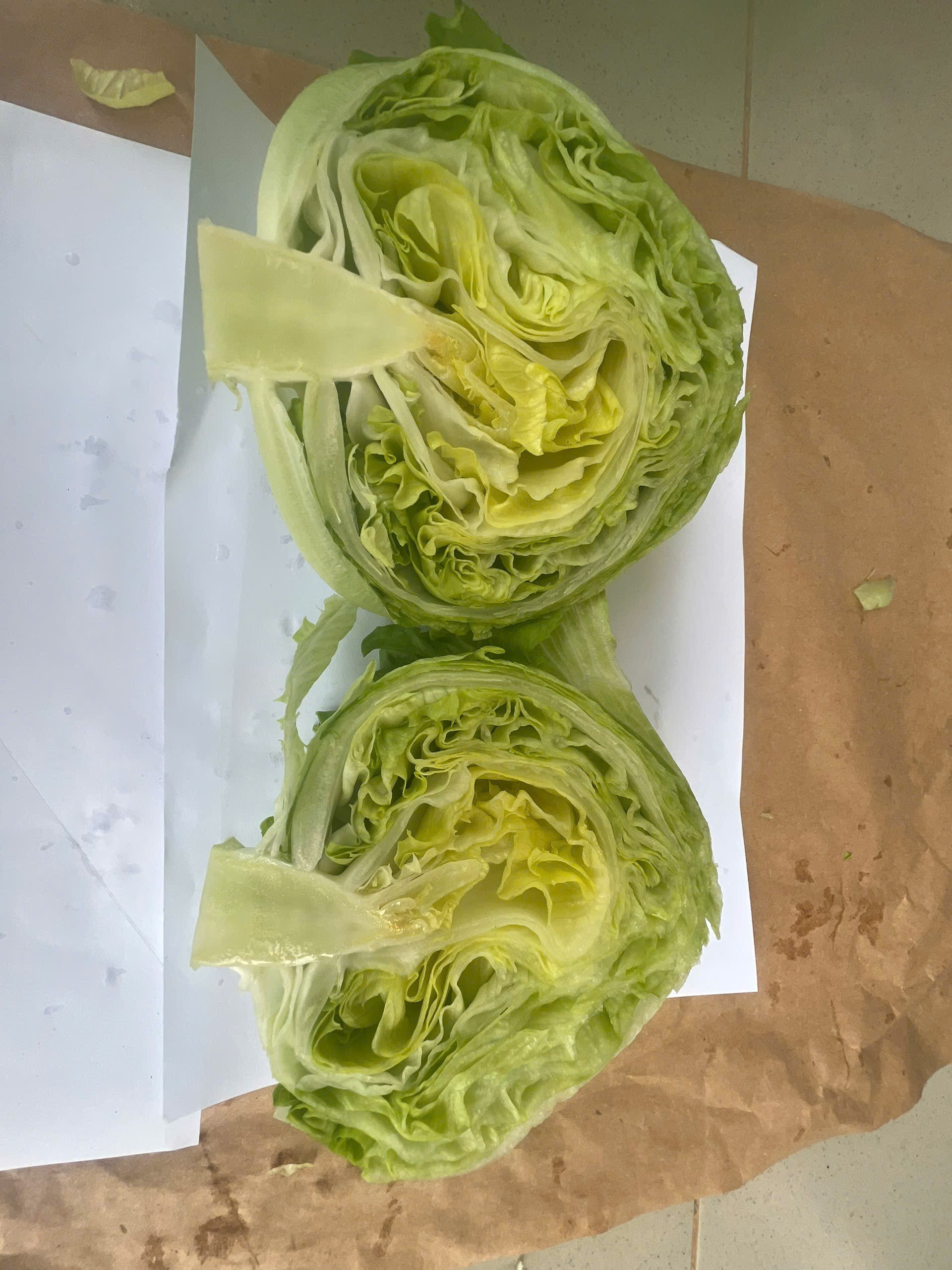 | 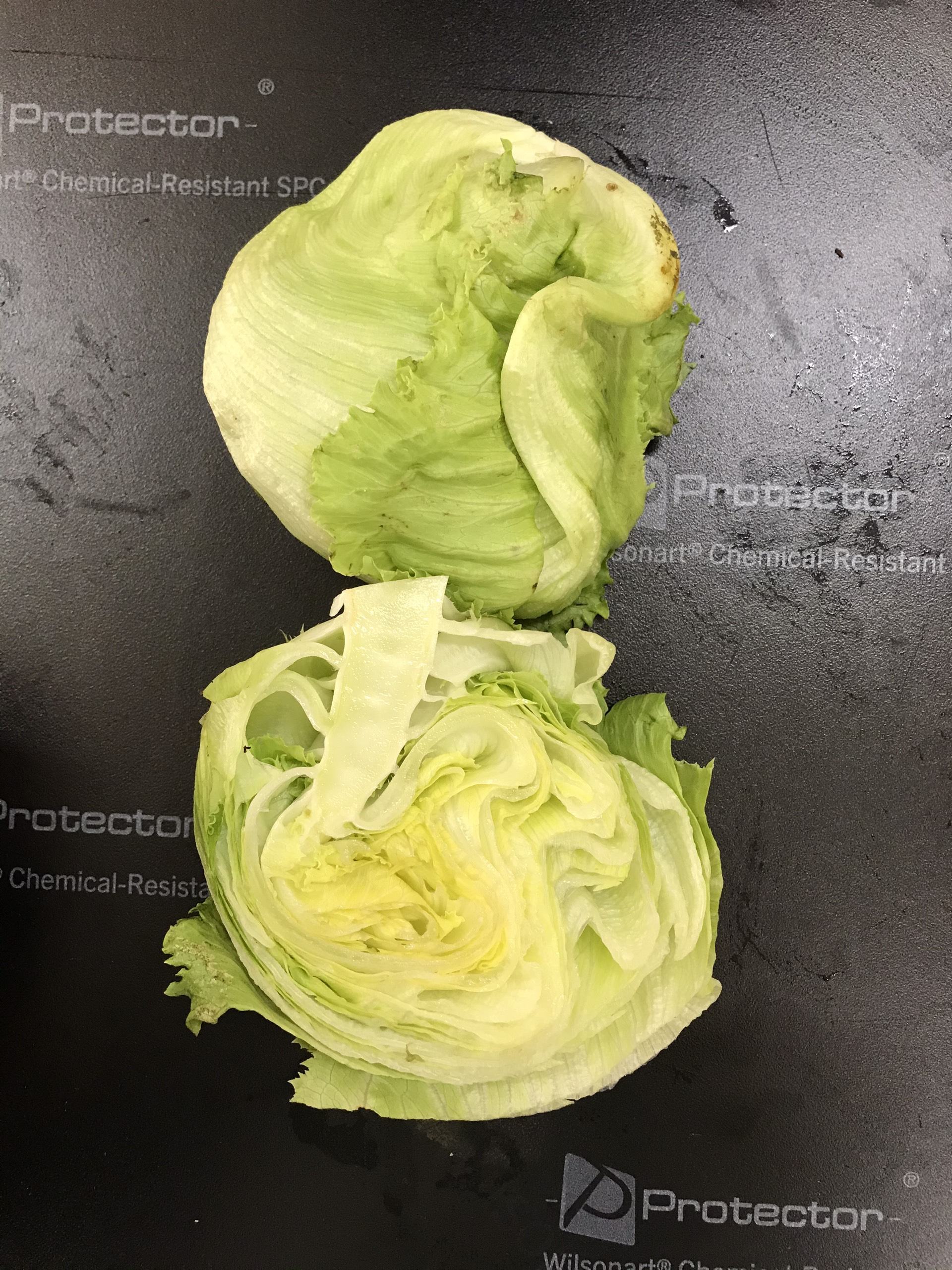 | 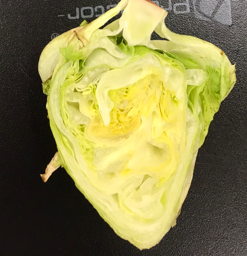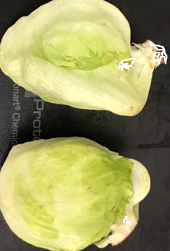 | 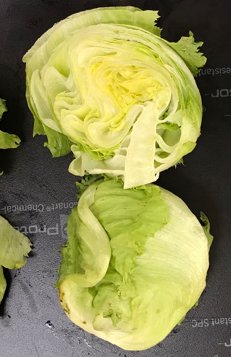 |
| 6 | 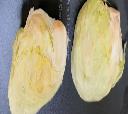 | 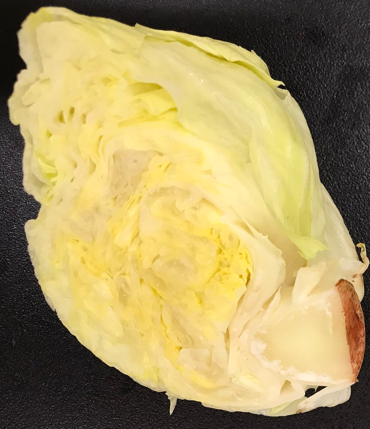  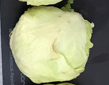 | 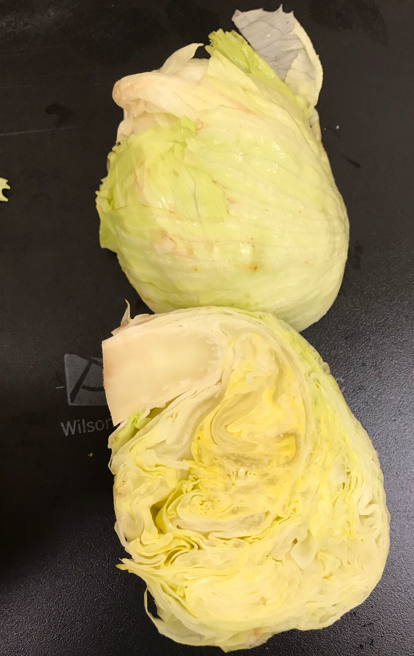 | 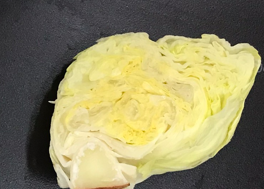  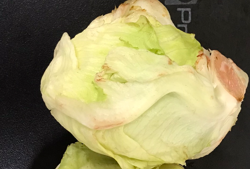 | 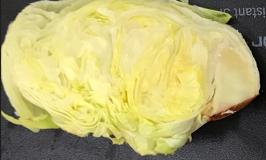  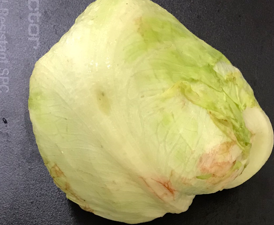 | 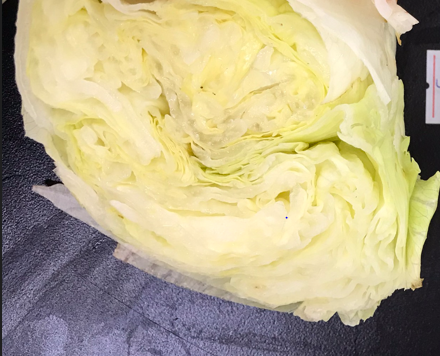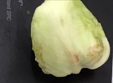 | 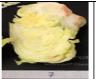  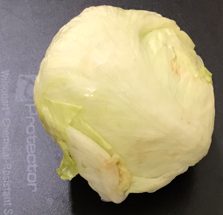 | 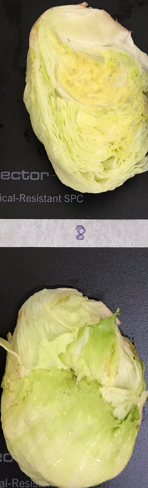 | 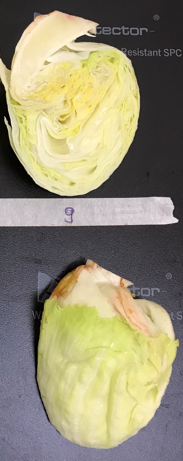 |
| 12 | 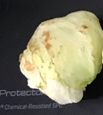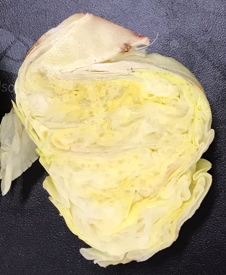 | 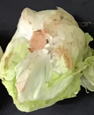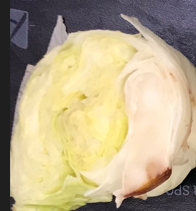 | 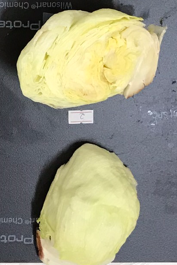 | 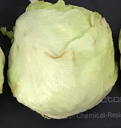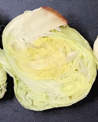 | 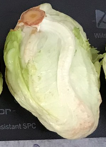  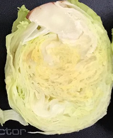 | 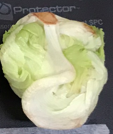  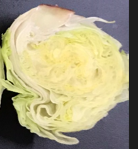 | 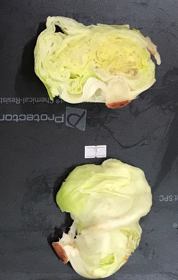 | 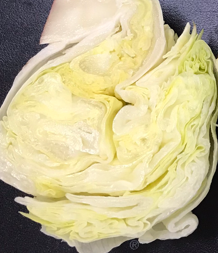  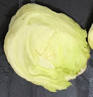 | 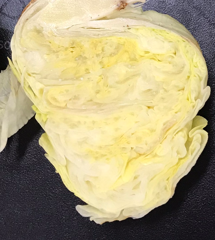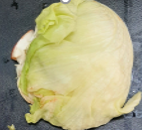 |
| 18 | 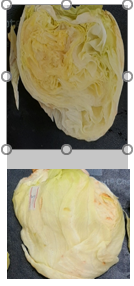 | 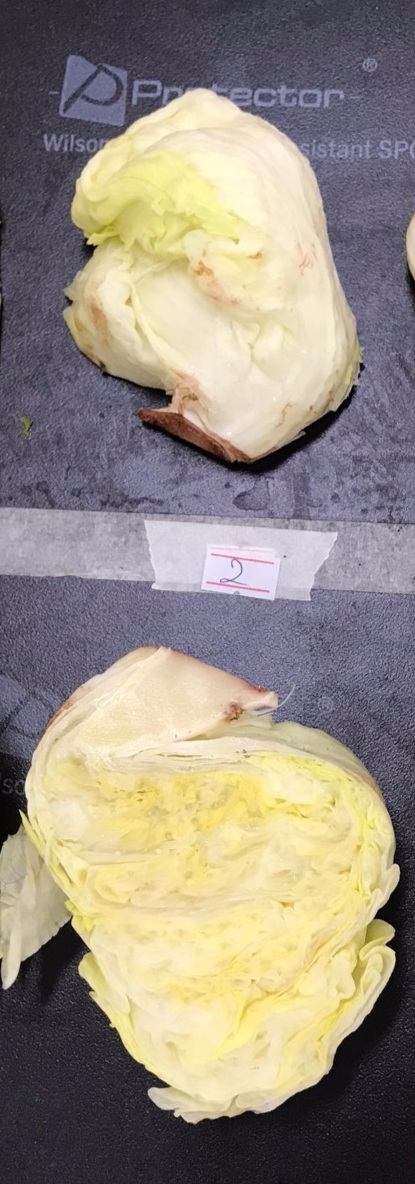 | 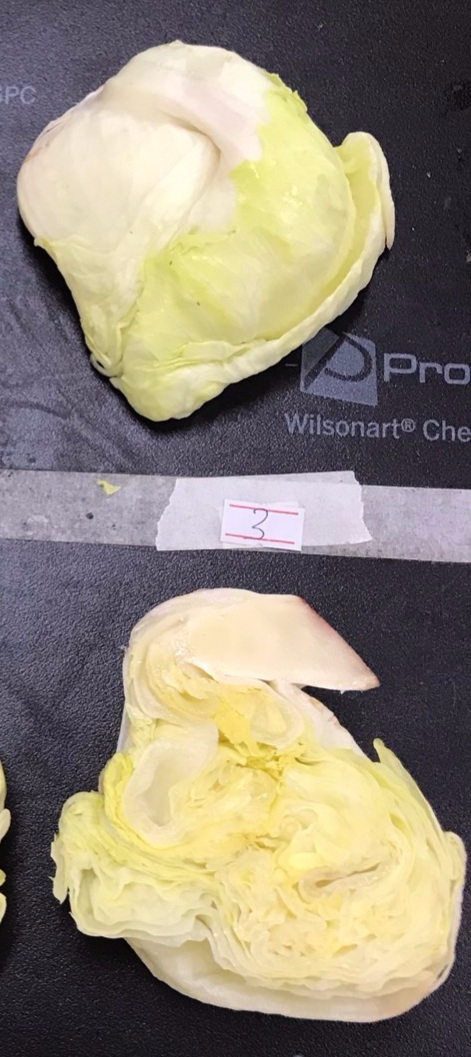 | 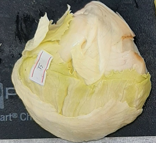  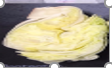 | 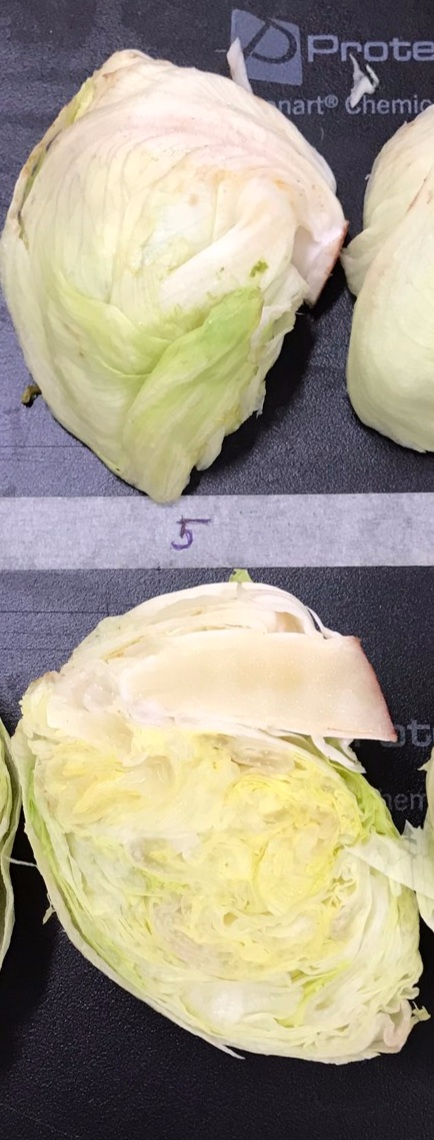 | 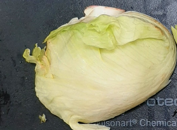  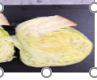 | 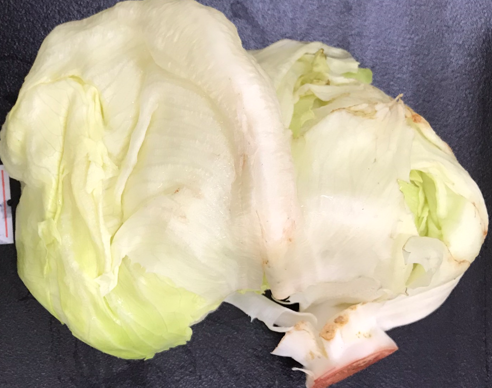  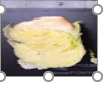 | 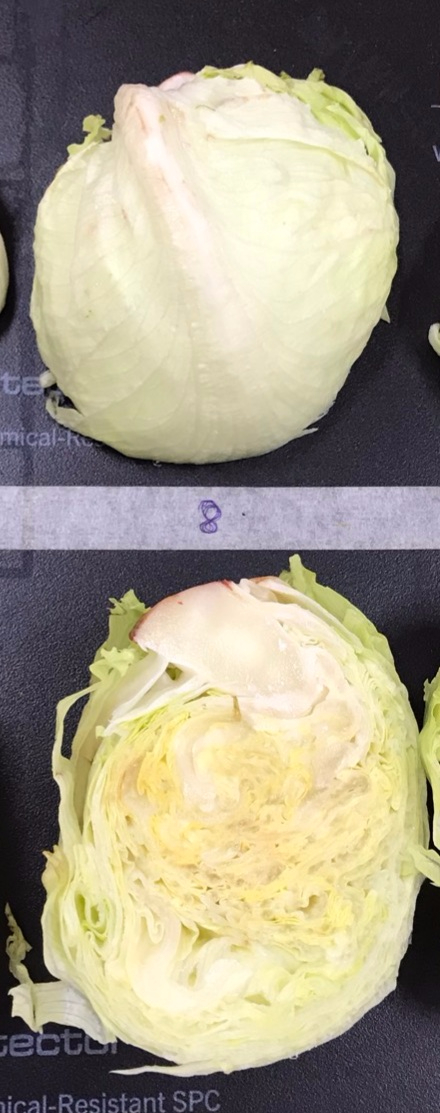 | 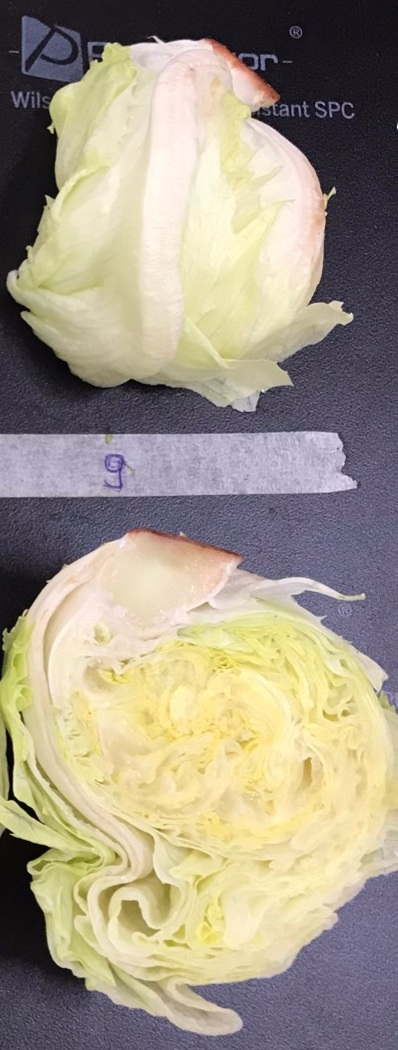 |
| 24 | 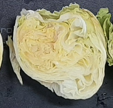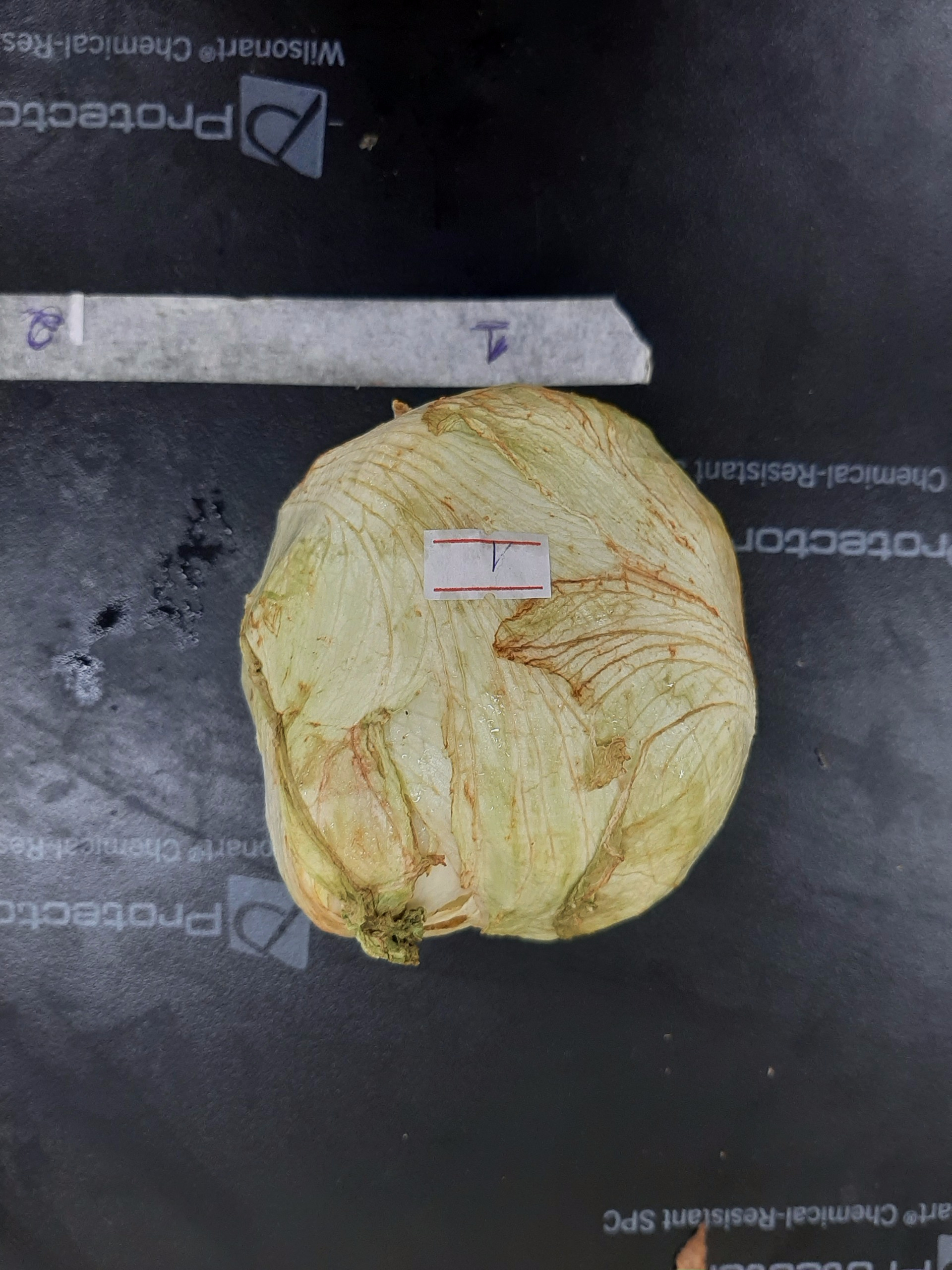 | 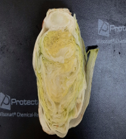  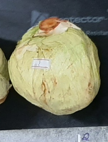 | 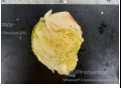  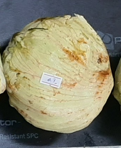 | 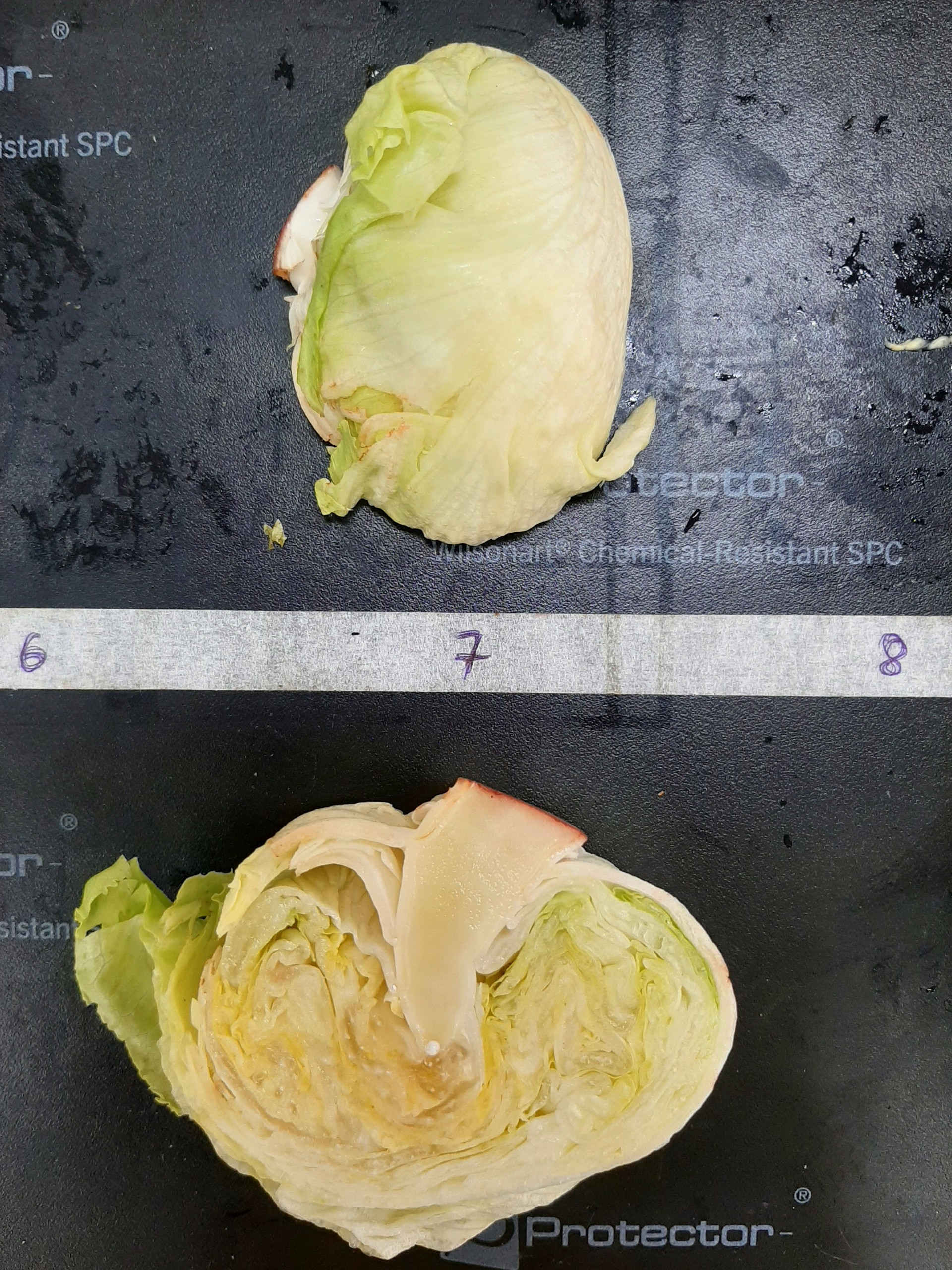 | 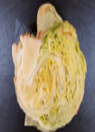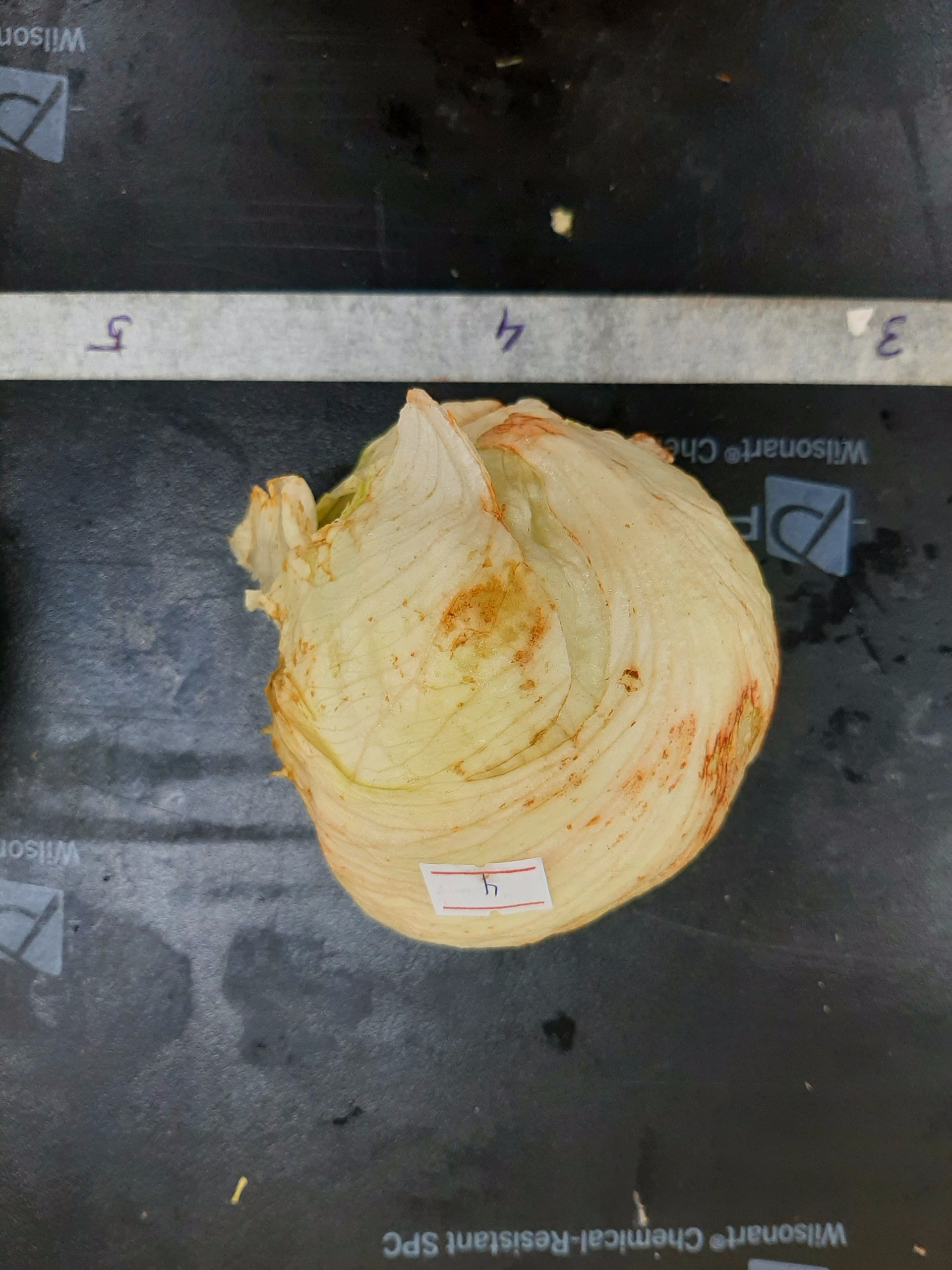 | 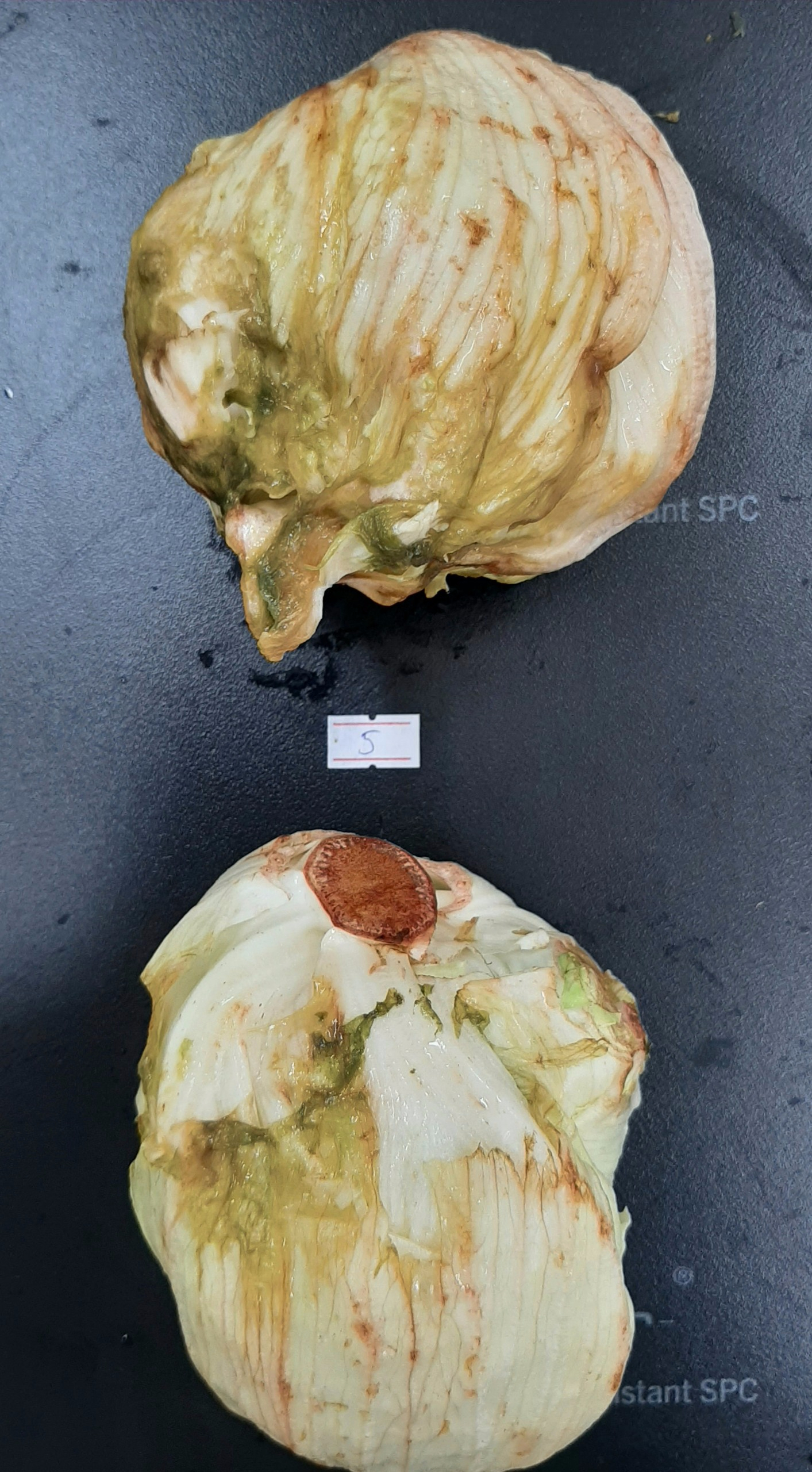 | 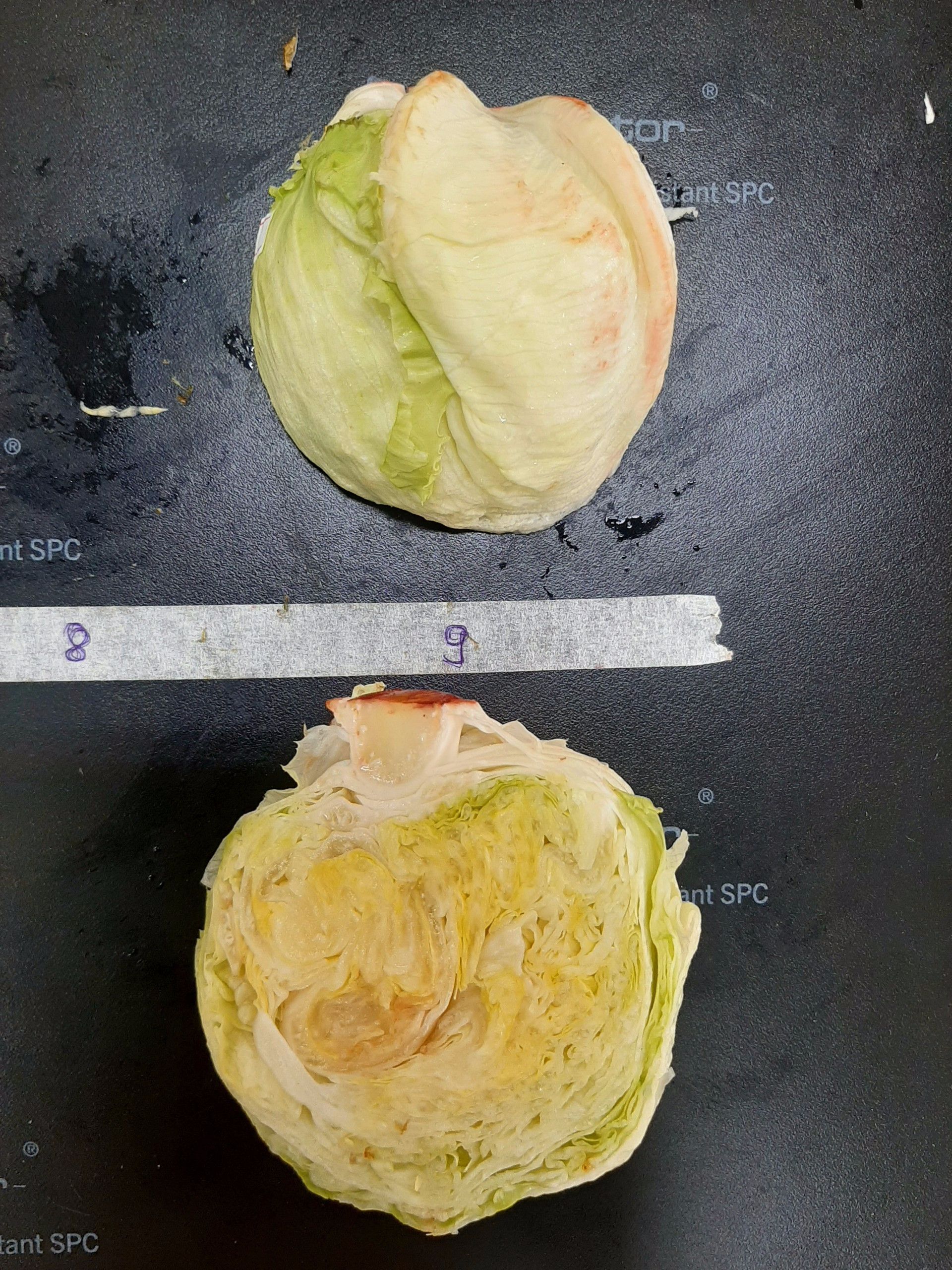 | 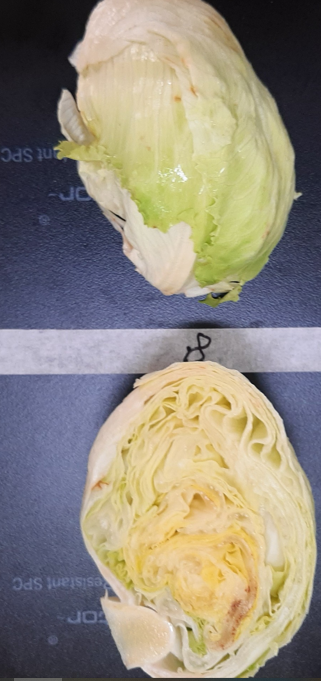 | 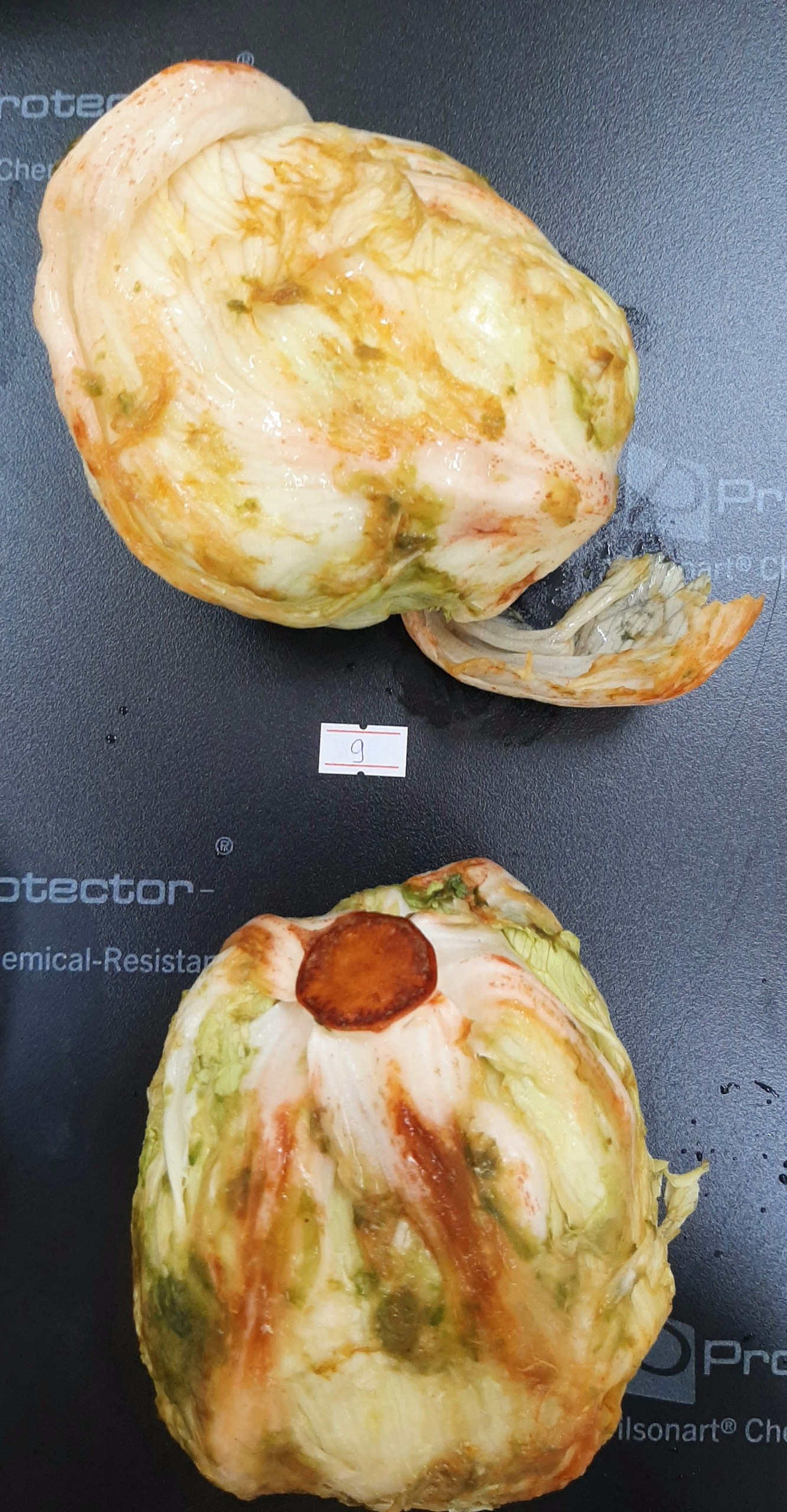 |
| 30 | 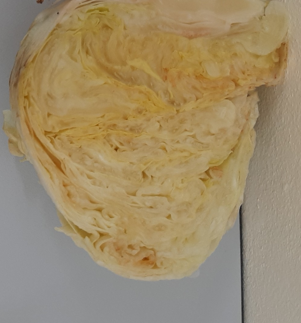  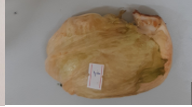 | 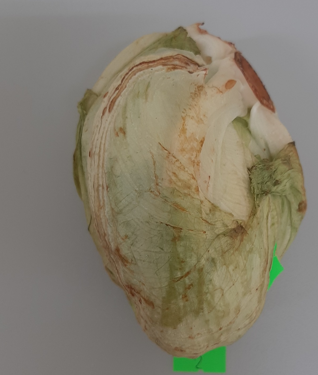  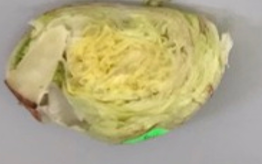 | 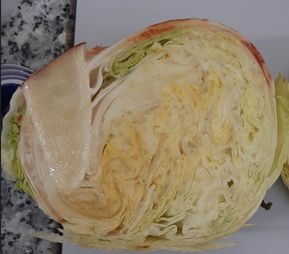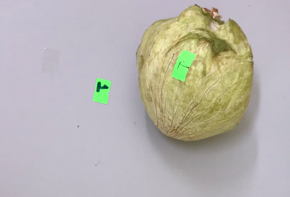 | 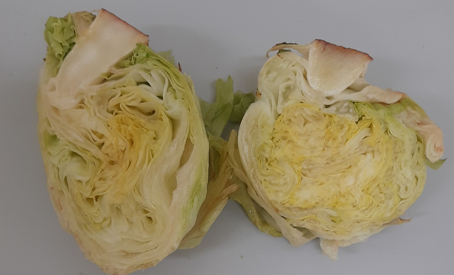  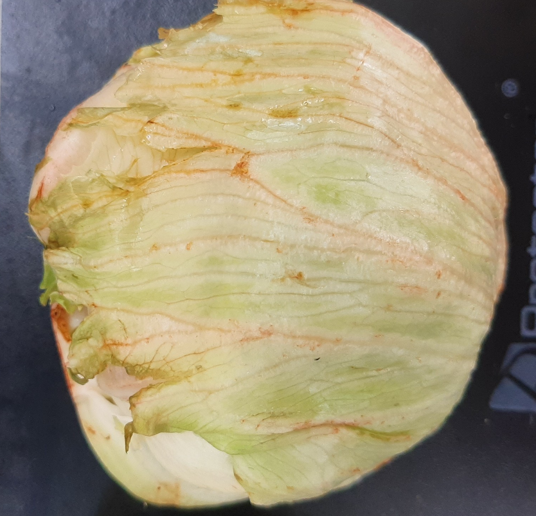 | 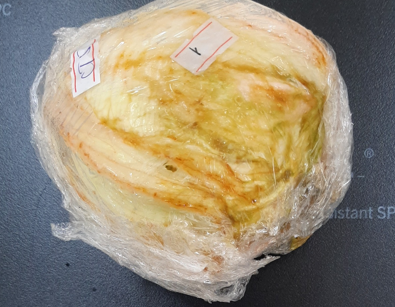  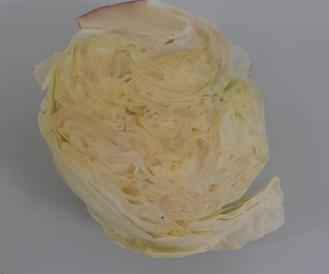 | 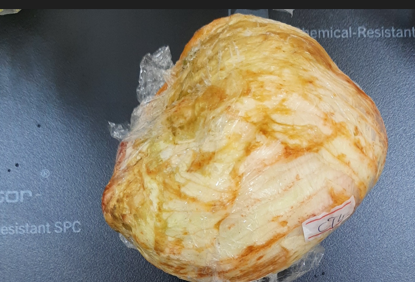  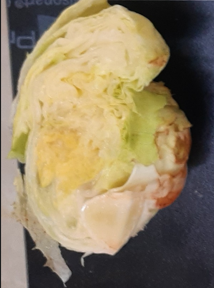 | 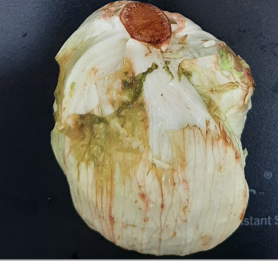  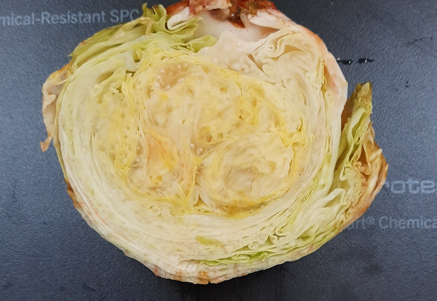 | 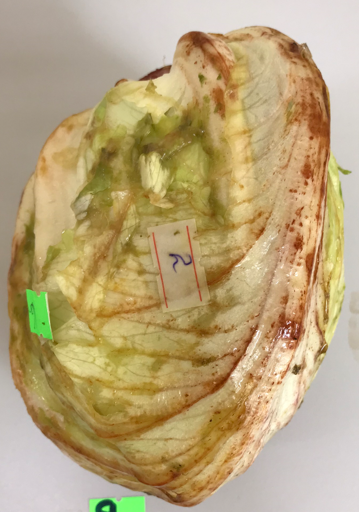  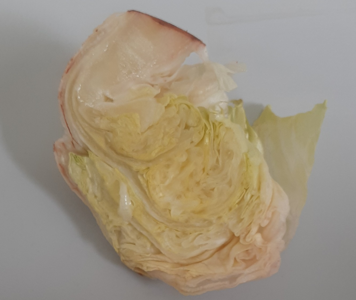 | 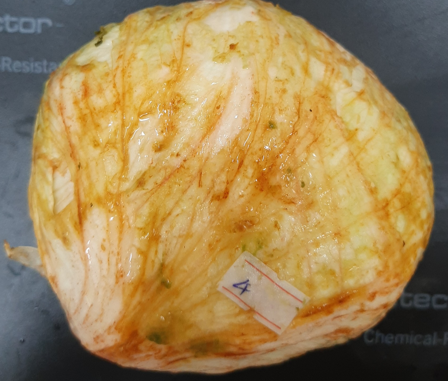  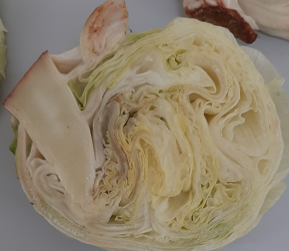 |
| 33 | 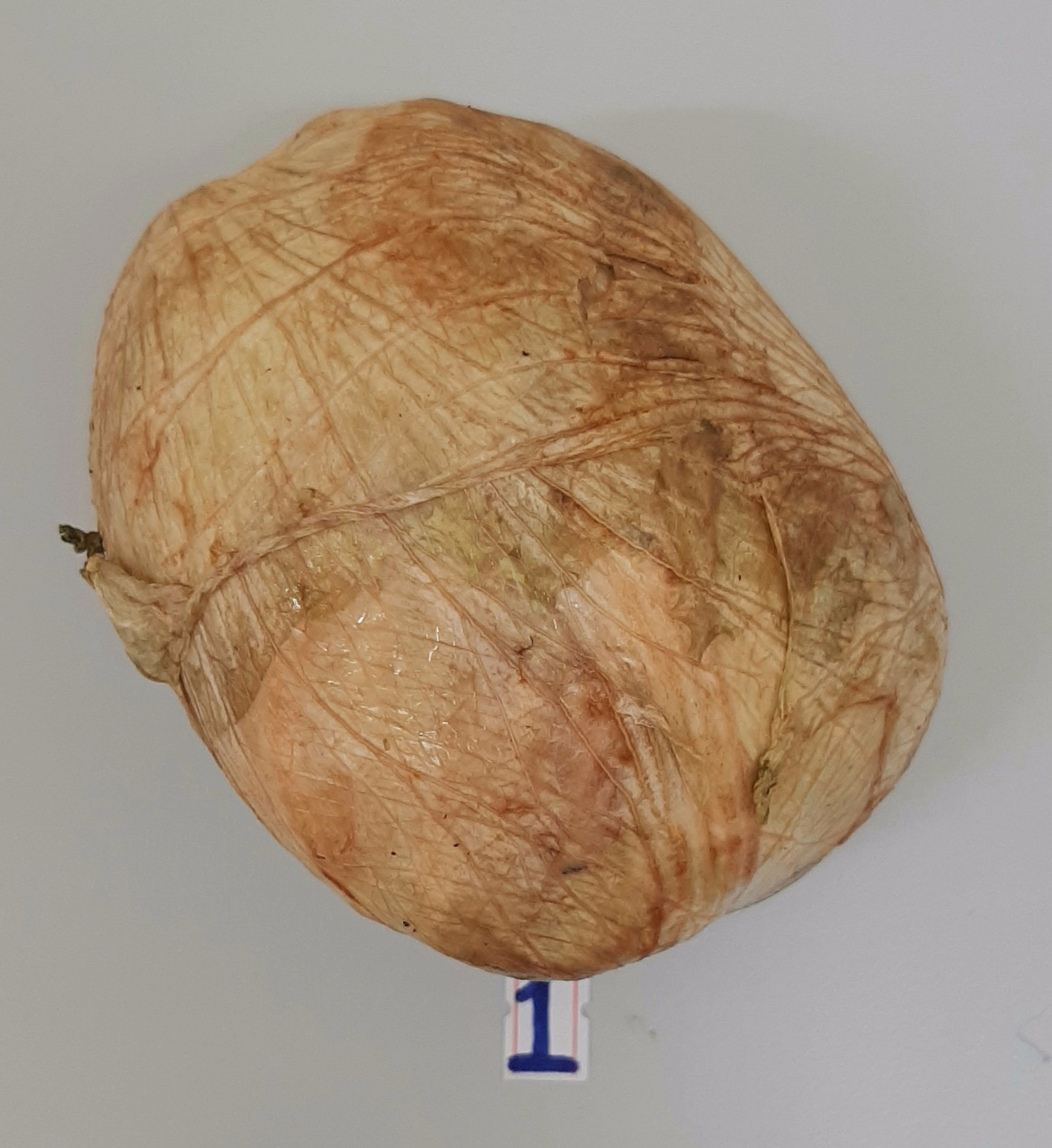  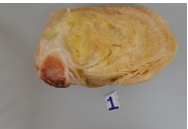 | 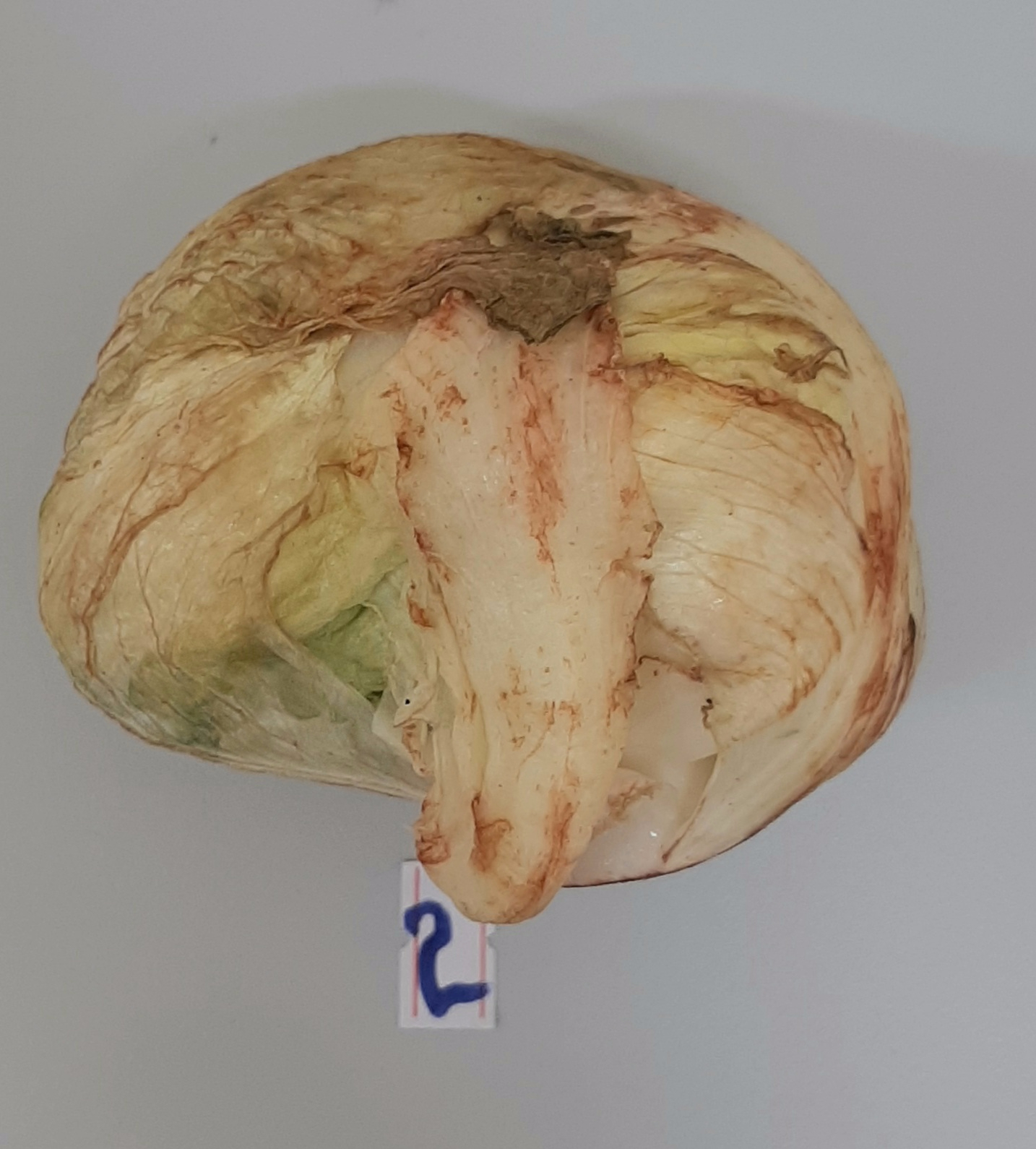  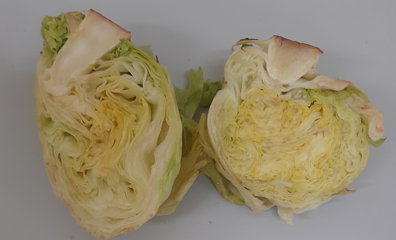 | 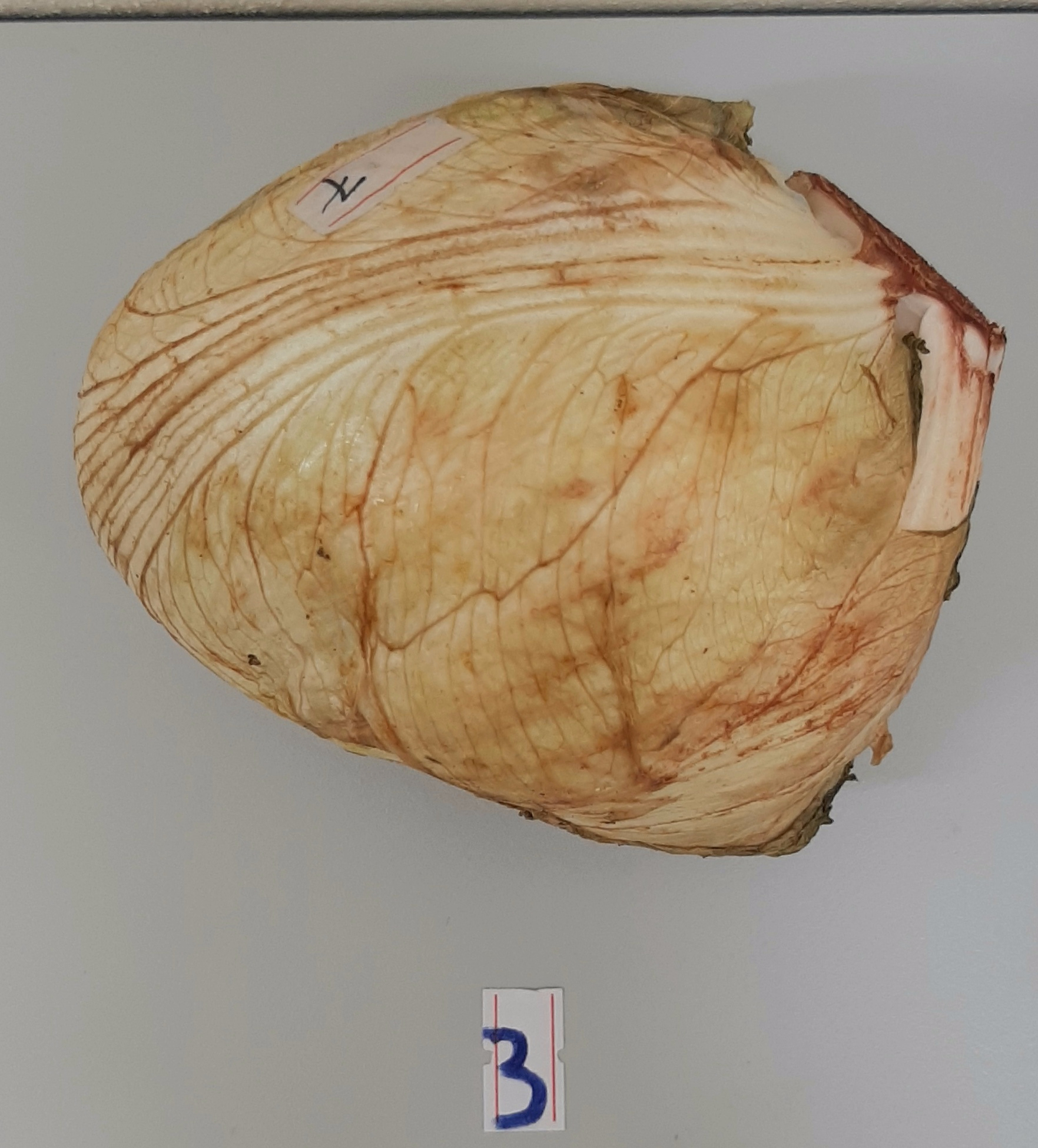  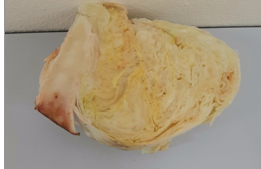 | 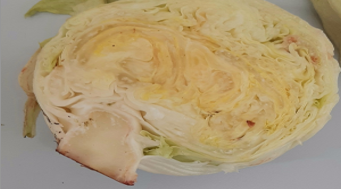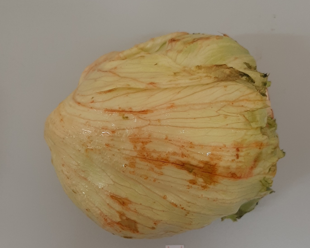 | 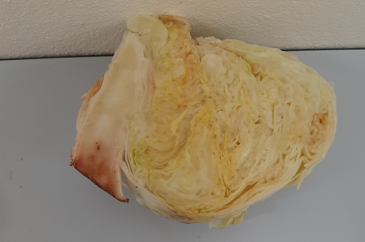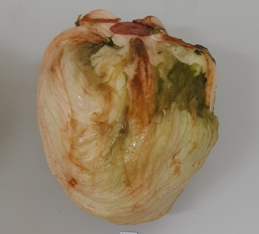 | 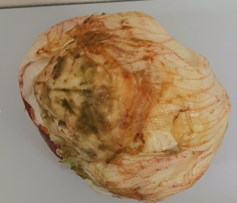  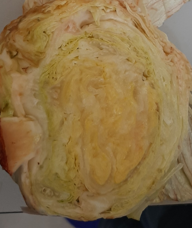 |  |  |  |

**Note**: F1=Without primary packaging + carton box ;F2=Without primary packaging + carton box is glued with LDPE ;F3=Without primary packaging + Danpla plastic box;F4= Wrapped by LDPE +Carton box; F5= Wrapped by LDPE+carton box is glued with LDPE;F6= Wrapped by LDPE+Danpla plastic box;F7= Wrapped by Green MAP+Carton box;F8= Wrapped by Green MAP+Carton box is glued with LDPE and F9= Wrapped by Green MAP+Danpla Plastic box.

**“F”** is stand for treatment combinatio
